# Tetherin/BST-2 Antagonism by Nef Depends on a Direct Physical Interaction between Nef and Tetherin, and on Clathrin-mediated Endocytosis

**DOI:** 10.1371/journal.ppat.1003487

**Published:** 2013-07-11

**Authors:** Ruth Serra-Moreno, Kerstin Zimmermann, Lawrence J. Stern, David T. Evans

**Affiliations:** 1 Division of Microbiology, New England Primate Research Center, Harvard Medical School, Southborough, Massachusetts, United States of America; 2 Department of Pathology, University of Massachusetts Medical School, Worcester, Massachusetts, United States of America; Fred Hutchinson Cancer Research Center, United States of America

## Abstract

Nef is the viral gene product employed by the majority of primate lentiviruses to overcome restriction by tetherin (BST-2 or CD317), an interferon-inducible transmembrane protein that inhibits the detachment of enveloped viruses from infected cells. Although the mechanisms of tetherin antagonism by HIV-1 Vpu and HIV-2 Env have been investigated in detail, comparatively little is known about tetherin antagonism by SIV Nef. Here we demonstrate a direct physical interaction between SIV Nef and rhesus macaque tetherin, define the residues in Nef required for tetherin antagonism, and show that the anti-tetherin activity of Nef is dependent on clathrin-mediated endocytosis. SIV Nef co-immunoprecipitated with rhesus macaque tetherin and the Nef core domain bound directly to a peptide corresponding to the cytoplasmic domain of rhesus tetherin by surface plasmon resonance. An analysis of alanine-scanning substitutions identified residues throughout the N-terminal, globular core and flexible loop regions of Nef that were required for tetherin antagonism. Although there was significant overlap with sequences required for CD4 downregulation, tetherin antagonism was genetically separable from this activity, as well as from other Nef functions, including MHC class I-downregulation and infectivity enhancement. Consistent with a role for clathrin and dynamin 2 in the endocytosis of tetherin, dominant-negative mutants of AP180 and dynamin 2 impaired the ability of Nef to downmodulate tetherin and to counteract restriction. Taken together, these results reveal that the mechanism of tetherin antagonism by Nef depends on a physical interaction between Nef and tetherin, requires sequences throughout Nef, but is genetically separable from other Nef functions, and leads to the removal of tetherin from sites of virus release at the plasma membrane by clathrin-mediated endocytosis.

## Author Summary

Tetherin (BST-2, CD317 or HM1.24) is an interferon-inducible cellular restriction factor that prevents the release of enveloped viruses from infected cells. Human and simian immunodeficiency viruses have evolved to use different viral proteins to overcome the anti-viral effects of tetherin. Whereas HIV-1 Vpu and HIV-2 Env counteract human tetherin, most SIVs use the accessory protein Nef to counteract tetherin in their non-human primate hosts. Here we show that the mechanism of tetherin antagonism by SIV Nef involves a direct physical interaction between the core domain of Nef and the cytoplasmic domain of tetherin, which results in the removal of tetherin from sites of virus assembly and release on the cell surface by a mechanism that depends on clathrin and dynamin 2. The Nef-mediated internalization of tetherin leads to the accumulation of tetherin within lysosomal compartments, suggesting that, similar to CD4− and MHC I-downregulation, Nef promotes the lysosomal degradation of tetherin.

## Introduction

Mammalian cells express a number of proteins that inhibit specific steps of virus replication. One such factor, tetherin (BST-2 or CD317), impairs the release of enveloped viruses from infected cells [1], [2], [3], [4], [5]. Tetherin is a type II integral membrane protein with a topology that allows both ends of the molecule to be anchored in lipid membranes [6]. It has an N-terminal cytoplasmic domain followed by a single-pass transmembrane domain, an extracellular coiled-coil domain and a C-terminal glycosyl-phosphatidylinositol (GPI) anchor [6]. Under conditions of interferon-induction, tetherin is upregulated and becomes incorporated into virus particles as they attempt to bud from infected cells [7], [8], [9]. Captured virions are then internalized and routed to lysosomal compartments for degradation by a mechanism that involves interactions between the cytoplasmic domain of tetherin and the endocytosis machinery of the cell [7], [10].

Tetherin has played an important role in shaping the course of lentiviral evolution in primates, having selected for at least three different viral gene products to overcome this restriction factor (reviewed in [11], [12]). Whereas HIV-1 Vpu and HIV-2 Env antagonize human tetherin [4], [5], [13], the majority of SIVs use Nef to counteract the tetherin proteins of their non-human primate hosts [14], [15], [16]. Indeed, HIV-1 Vpu and HIV-2 Env appear to have acquired the ability to antagonize tetherin due to the absence of sequences in the cytoplasmic domain of human tetherin that confer susceptibility to Nef [11], [12].

Alternative models have been proposed for the mechanism of tetherin antagonism by HIV-1 Vpu. Vpu physically associates with tetherin via membrane-spanning domain interactions [17], [18], [19], [20], recruits ßTrCP-2, a component of the Skp1-Cullin1-F-box ubiquitin ligase complex, promoting the ubiquitination of non-lysine residues in the cytoplasmic domain of tetherin [21], [22], and uses the ESCRT-mediated trafficking of tetherin [23] for degradation in lysosomes [17], [19], [24], [25], [26]. There is also evidence that Vpu may antagonize tetherin in the absence of degradation by sequestering the protein in a perinuclear compartment, either by retaining newly synthesized tetherin, or by blocking the recycling of tetherin to the plasma membrane [24], [27], [28], [29]. Vpu-mediated downmodulation of tetherin and enhancement of virus release were also recently shown to be dependent in part on clathrin-mediated endocytosis [30].

The mechanism of tetherin antagonism by HIV-2 Env depends on a physical interaction between Env and tetherin, and a conserved tyrosine-based endocytosis motif in the cytoplasmic tail of gp41 [13], [31], [32]. The sequences required for Env interactions with tetherin are poorly defined, but appear to reside in the extracellular domains of both proteins, as indicated by analyses of recombinant forms of Env and tetherin [13], [33], and the identification of substitutions in the ectodomains of each protein that disrupt tetherin antagonism [32], [33], [34], [35]. HIV-2 Env does not promote the degradation of tetherin, but leads to the internalization and sequestration of tetherin by a clathrin-dependent mechanism, consistent with the trapping of tetherin in recycling endosomes [13], [30], [31].

Comparatively little is known about the mechanism of tetherin antagonism by Nef. The Nef proteins of phylogenetically diverse SIVs, including SIV_smm/mac_, SIV_agm_ and SIV_cpz_, antagonize the tetherin proteins of their non-human primate hosts, but not human tetherin [14], [15], [16]. This specificity maps to a five amino acid sequence that is present in the cytoplasmic tails of non-human primate tetherin proteins (G/D_14_DIWK_18_ in rhesus macaques, sooty mangabeys and chimpanzees), but absent from the corresponding region of human tetherin [15], [16]. We previously reported that SIV Nef downregulates rhesus tetherin from the surface of transfected and infected cells [15], [36]. Zhang et al. further demonstrated that this activity is AP-2-dependent [37]. Here we demonstrate a direct physical interaction between SIV Nef and rhesus tetherin, define residues throughout Nef required for tetherin antagonism, and demonstrate that the anti-tetherin activity of Nef is dependent, at least in part, on clathrin-mediated endocytosis.

## Results

### SIV Nef binds selectively to the cytoplasmic domain of rhesus tetherin

**Figure 1:**
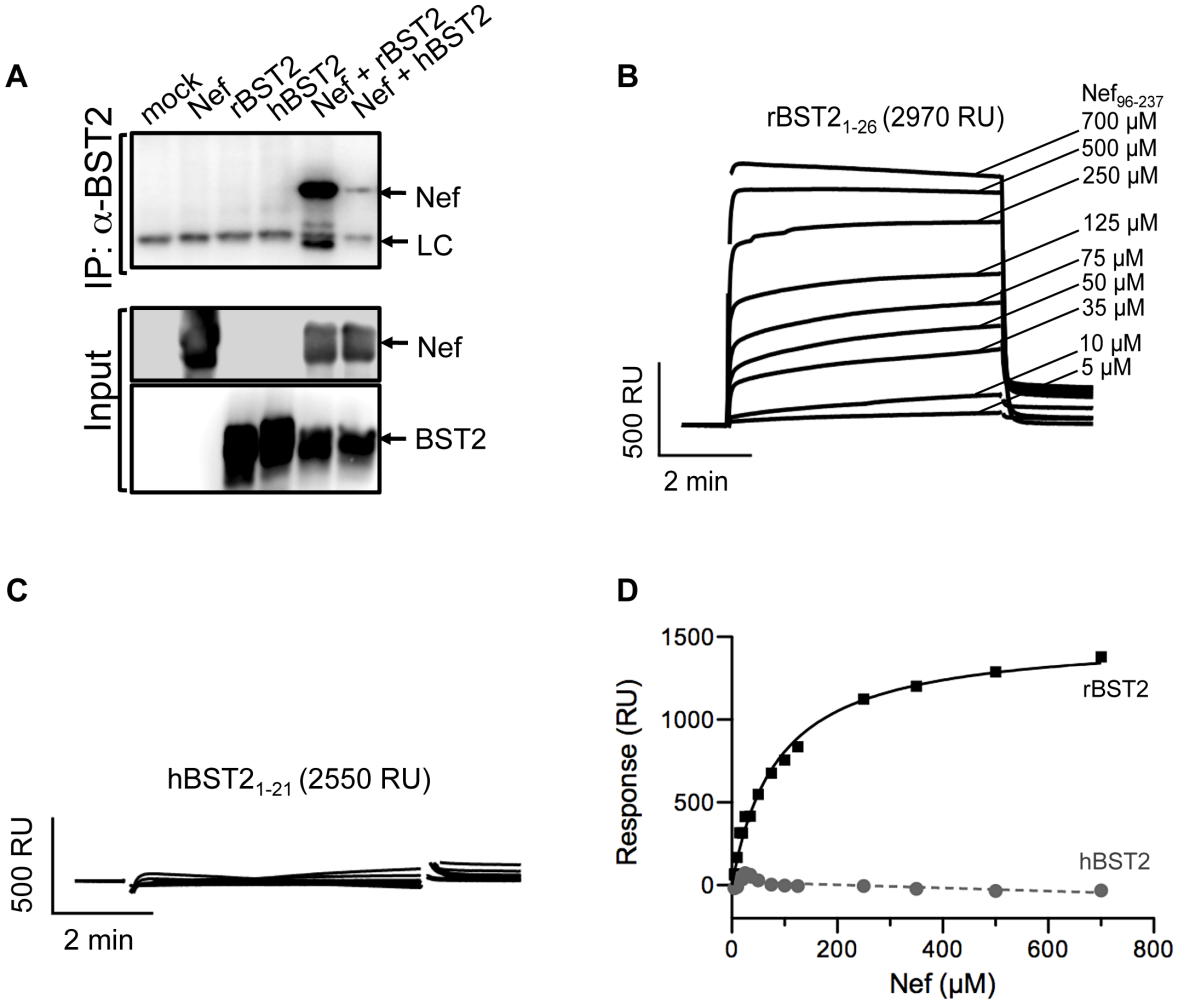
SIV Nef physically interacts with the cytoplasmic domain of rhesus tetherin. (A) 293T cells were co-transfected with constructs expressing SIV Nef and either rhesus tetherin (rBST-2), human tetherin (hBST-2) or empty vector. Cell lysates were immunoprecipitated with a monoclonal antibody to tetherin, proteins were separated by SDS-PAGE and transferred to PVDF membranes. Western blots were developed with a monoclonal antibody to SIV Nef. Western blots were also performed for whole cell lysates to determine the input levels of these proteins. (B & C) Surface plasmon resonance (SPR) analyses were performed to test for direct binding between Nef and rhesus tetherin or human tetherin. Peptides corresponding to the cytoplasmic domain of rBST-2 and hBST-2 were biotinylated at conserved cysteines and captured on neutravidin-coated CM5 sensor chips. The binding of Nef to tetherin was assessed by flowing purified recombinant SIV_mac_239 Nef_4–263_ and Nef_96–237_ proteins over the peptide-coated biosensor chips. (D) The dissociation constant and maximum response rate for this interaction were determined by equilibrium analysis (see materials and methods for details).

SIV Nef was tested for a physical interaction with tetherin by co-immunoprecipitation. Tetherin was immunoprecipitated from lysates of 293T cells co-transfected with constructs expressing Nef and either human or rhesus macaque tetherin. Immunoprecipitated proteins were separated by SDS-PAGE, and western blots were probed with monoclonal antibodies to Nef and to tetherin. In accordance with the selective activity of Nef in opposing restriction by tetherin [15], [16], SIV Nef strongly co-immunoprecipitated with rhesus tetherin, but not with human tetherin (Figure 1A).

To determine if this interaction is direct, SIV Nef was tested for binding to peptides corresponding to the N-terminal cytoplasmic domains of rhesus and human tetherin by surface plasmon resonance (SPR). Tetherin peptides were biotinylated at conserved cysteine residues (C_25_ in rBST-2 and C_20_ in hBST-2) and coupled to the surface of neutravidin-coated CM5-BIAcore chips to mimic the native orientation of the N-terminus of tetherin on the inner leaflet of the plasma membrane. Recombinant SIV_mac_239 Nef proteins containing residues 4–263 (Nef_4–263_) and 96–237 (Nef_96–237_) were flowed over the immobilized peptides to assess binding. SIV Nef_96–237_ bound to the N-terminal peptide of rhesus tetherin (Figure 1B), but not to the corresponding peptide of human tetherin (Figure 1C). The dissociation constant and the maximum response for SIV Nef_96–237_ binding to rhesus tetherin were determined by equilibrium analysis (K_d.app_ 401+/−114 µM) (Figure 1D). The nearly full-length Nef protein, Nef_4–263_, also bound to rhesus tetherin (data not shown). However, the K_d_ of this interaction could not be determined due to artifacts at protein concentrations greater than 300 µM that may reflect Nef dimerization. These results reveal a direct physical interaction between SIV Nef and the cytoplasmic domain of rhesus macaque tetherin.

### Identification of SIV Nef residues required for tetherin antagonism

**Figure 2:**
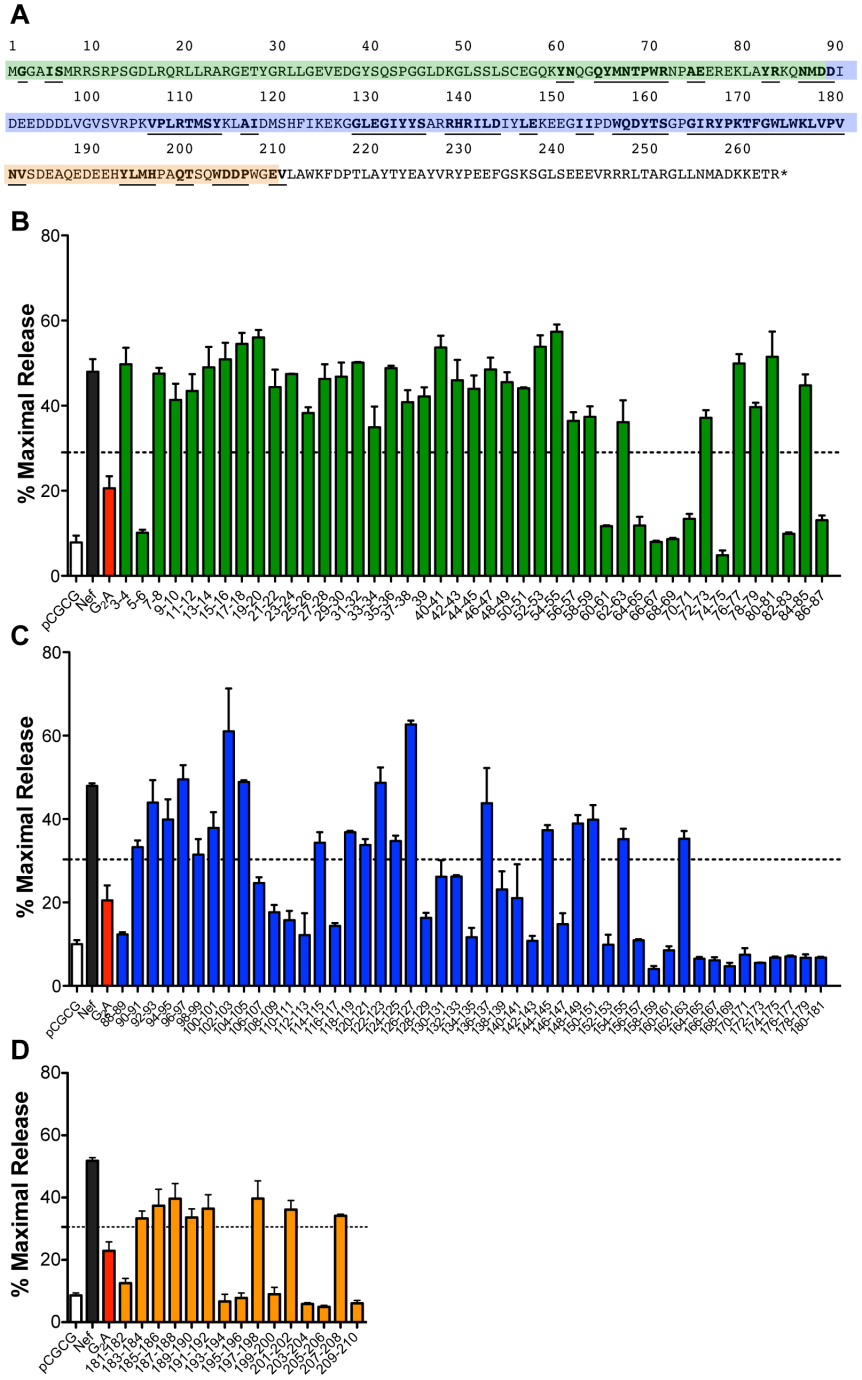
Identification of residues in SIV Nef necessary for tetherin antagonism. (A) Predicted amino acid sequence of SIV_mac_239 Nef. The highlighted sequences correspond to the N-terminal domain (green), the globular core (blue), and the flexible loop region (orange). Substitutions in residues that impaired tetherin antagonism are underlined and in bold. (B, C and D) 293T cells were co-transfected with SIV Δ*nef* proviral DNA together with constructs expressing rhesus tetherin and either wild-type or mutant Nef proteins. The percentage of virus release was determined by measuring the accumulation of SIV p27 in the culture supernatant in the presence of tetherin relative to transfections with empty vector. Controls include virus release in the absence of Nef (pCGCG; white), wild-type Nef (black) and Nef with a glycine-to-alanine substitution in the myristoylation site (G_2_A; red). Substitutions in the N-terminal domain are indicated by green bars (B), substitutions in the globular core domain are indicated by blue bars (C), and substitutions in the flexible loop region are indicated by orange bars (D). Error bars represent the standard deviation of duplicate transfections and the dotted line indicates 3 standard deviations over the activity of the G_2_A mutant.

To identify sequences in SIV Nef that contribute to tetherin antagonism, 103 pair-wise alanine-scanning substitutions were introduced throughout the N-terminal, globular core and the flexible loop regions of SIV_mac_239 Nef (residues 3–210), and these mutants were tested for their ability to counteract rhesus tetherin in virus release assays (Figure 2). Mutations in the C-terminal domain (residues 211–263) were not tested, since these sequences can be deleted without affecting the anti-tetherin activity of Nef (data not shown). Virus release for 43 of the Nef mutants was reduced to a similar or greater extent than a myristoylation site mutant (G_2_A), which was previously shown to impair tetherin antagonism [15]. These results were corroborated by western blot analyses comparing p55 Gag expression in cell lysates to the accumulation of p27 capsid (CA) in the cell culture supernatant (Figure S1). This approach identified 9 substitutions in the N-terminal domain (Figure 2B), 27 substitutions in the globular core domain (Figure 2C), and 7 substitutions in the flexible loop region of Nef (Figure 2D) that disrupt tetherin antagonism.

### Identification of residues that contribute to the interaction between Nef and tetherin

**Figure 3:**
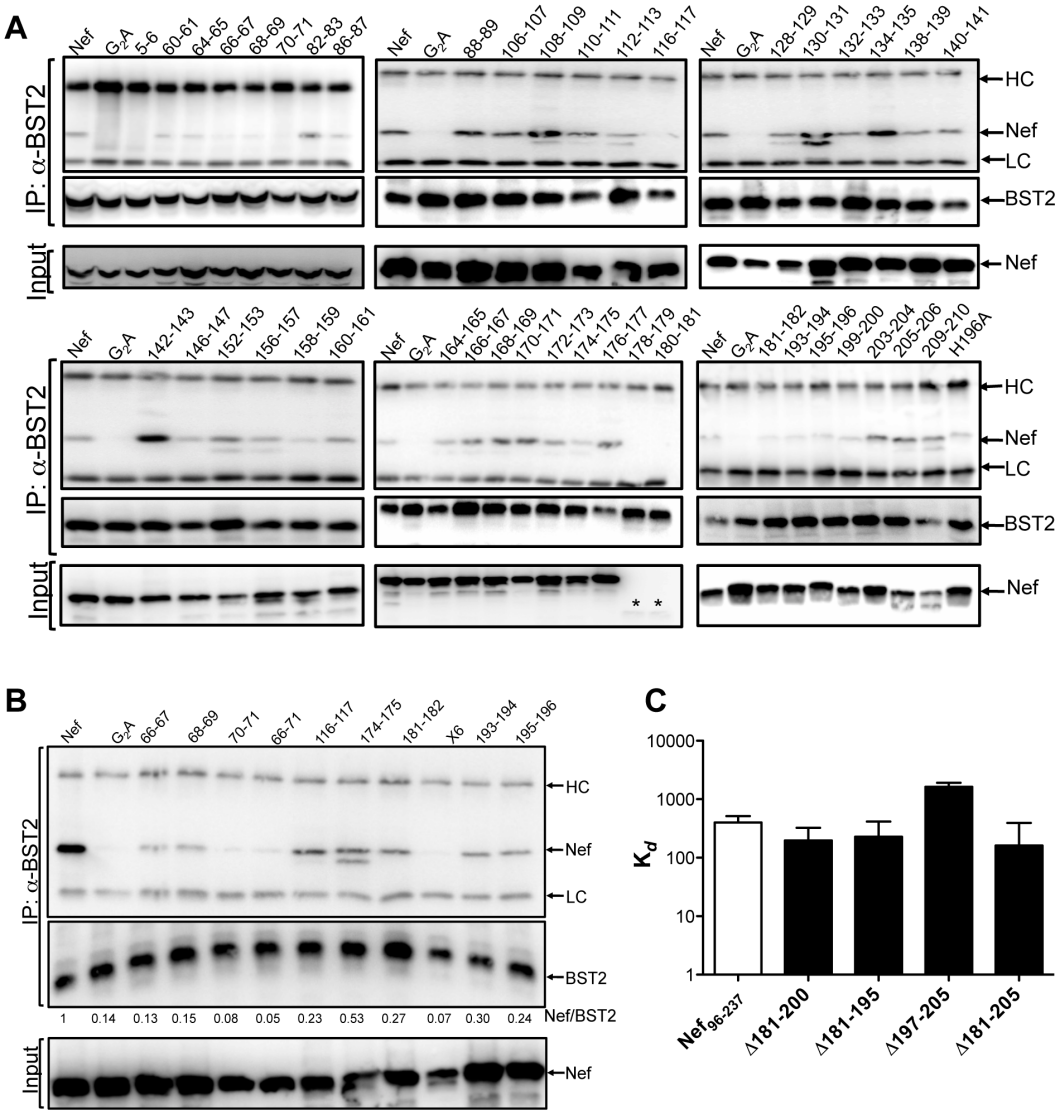
Identification of residues in SIV Nef that contribute to interactions with tetherin. (A) Co-immunoprecipitation assays with the Nef mutants that impair anti-tetherin activity were performed to identify residues that diminish binding to tetherin. 293T cells were co-transfected with constructs expressing rhesus tetherin, and either wild-type or mutant Nef proteins. Cell lysates were immunoprecipitated using a monoclonal antibody to tetherin, and blots were probed with monoclonal antibodies to SIV Nef and tetherin. (B) Combinations of alanine substitutions in Nef that were shown to impair binding to rhesus tetherin in panel A were tested in additional co-immunoprecipitation assays as described above. (C) Estimated K_d.app_ values were determined for the binding of SIV_mac_239 Nef_96–237_ with the indicated deletions in the flexible loop region to the cytoplasmic domain of rhesus tetherin by SPR. Bands corresponding to the antibody heavy and light chain are indicated (HC and LC). Asterisks indicate the absence of detectable Nef protein.

To define residues in SIV Nef that contribute to interactions with tetherin, Nef mutants lacking anti-tetherin activity were tested for binding to rhesus tetherin by co-immunoprecipitation (Figure 3A). The ratios of the band intensities for Nef and tetherin in immunoprecipitates were calculated to quantify differences in binding to tetherin (Table 1). Substitutions at positions 2, 5–6, 66–67, 68–69 and 70–71 in the N-terminal domain, positions 116–117 and 174–175 in the globular core domain, and positions 181–182, 193–194, 195–196 and 199–200 in the flexible loop region diminished the co-immunoprecipitation of Nef with tetherin (Figure 3A, 3B and Table 1). Some of the substitutions in the globular core, particularly at positions 178–179 and 180–181, resulted in reduced levels of Nef protein in cell lysates (Figure 3A and Table 1). Hence, the loss of tetherin binding in these instances may reflect decreased Nef protein stability or expression rather than a disruption of tetherin contact residues. However, many of the core domain mutations that decreased steady-state levels of Nef did not result in a corresponding decrease in binding to tetherin, and a few paradoxically appear to have increased the stability of this interaction (Figure 3A and Table 1). Since many of these mutations did not completely abrogate binding to tetherin, combinations of alanine substitutions were also tested. The co-immunoprecipitation of Nef with tetherin was reduced to nearly undetectable levels by combining substitutions in the N-terminal domain, either alone (residues 66–71), or together with the substitutions in the globular core and flexible loop (x6: residues 66–71, 116–117, 174–175 and 181–182) (Figure 3B).

**Table 1:** Summary of the properties of the SIV Nef mutants lacking anti-tetherin activity.

	% BST2 antagonism^a^	BST2 binding^b^	Nef levels^c^	% CD4 expression^d^	% MHC I expression^e^	Relative infectivity^f^
**Nef**	100	1	***1***	***37.13***	***41.40***	***1***
**G_2_A**	32.04	0.16	1.02	79.43	93.57	0.81^**^
**5–6**	21.43	0.18	1.18	60.76	91.70	0.79^**^
**60–61**	24.77	0.70	1.22	71.95	38.73^**^	0.96^**^
**64–65**	25.09	0.54	1.18	71.54	32.44^**^	0.39
**66–67**	16.94	0.18	1.07	76.49	31.18^**^	0.43
**68–69**	18.36	0.14	1.05	73.93	30.12^**^	0.27
**70–71**	28.41	0.28	1.37	75.57	28.71^**^	0.69^**^
**74–75**	10.27	0.71	1.44	83.75	81.31	1.01^**^
**82–83**	21.00	1.38	0.44	72.13	***22.40*** ^**^	0.51
**86–87**	27.68	0.47	0.52	42.33^**^	29.32^**^	0.79^**^
**88–89**	26.91	0.93	1.12	76.28	38.57^**^	0.76^**^
**106–107**	53.63	0.82	***1.23***	39.61^**^	47.24^**^	0.87^**^
**108–109**	38.43	1.87	0.82	79.18	89.30	0.76^**^
**110–111**	34.26	1.29	0.75	65.06	105.52	0.91^**^
**112–113**	26.48	0.35	0.72	62.81	95.97	0.59
**116–117**	31.34	0.16	0.56	102.68	118.09	0.80^**^
**128–129**	35.45	1.11	0.70	***51.97*** ^*^	86.41	1.33^**^
**130–131**	46.05	2.40	0.42	58.22	91.15	1.29^**^
**132–133**	40.73	0.79	0.53	64.43	49.67^**^	0.80^**^
**134–135**	25.34	2.58	1.1	113.12	117.88	0.77^**^
**138–139**	50.33	0.84	0.97	***51.51*** ^*^	60.97	0.66^**^
**140–141**	45.83	1.38	0.71	60.06	56.69^**^	0.72^**^
**142–143**	23.44	5.10	0.60	95.17	111.06	***0.79*** ^**^
**146–147**	32.24	1.56	0.52	105.17	104.02	0.50
**152–153**	21.50	1.68	0.62	97.31	103.37	1.1^**^
**156–157**	23.80	1.46	0.86	110.99	100.27	0.85^**^
**158–159**	8.83	0.76	0.60	78.49	87.17	0.70^**^
**160–161**	18.60	1.64	0.58	55.78^*^	93.18	1.05^**^
**164–165**	14.13	1.34	0.63	82.35	97.46	0.74^**^
**166–167**	13.40	1.40	0.33	83.53	113.26	0.88^**^
**168–169**	10.24	2.10	0.33	86.10	88.91	0.54
**170–171**	16.29	2.61	0.35	54.84^*^	92.21	0.67^**^
**172–173**	12.08	1.34	0.40	81.75	98.37	1.06^**^
**174–175**	14.82	0.52	0.2	87.02	93.68	0.72^**^
**176–177**	15.34	3.17	0.27	63.49	103.43	0.74^**^
**178–179**	12.5	0.17	0.05	101.49	95.05	0.39
**180–181**	10.97	0.02	0.04	102.40	92.77	0.43
**181–182**	27.99	0.23	***1.11***	29.77^**^	34.02^**^	0.86^**^
**193–194**	14.86	0.22	***1.35***	49.56^**^	32.95^**^	0.80^**^
**195–196**	17.45	0.31	1.13	68.21	***36.54*** ^**^	0.42
**199–200**	20.06	0.43	***1.22***	38.48^**^	30.86^**^	0.74^**^
**203–204**	13.03	0.74	1.34	68.55	26.83^**^	0.93^**^
**205–206**	11.01	0.94	1.40	75.20	38.95^**^	0.84^**^
**209–210**	13.55	1.40	***1.07***	52.63^*^	55.94^*^	0.66^**^

a The percentage of BST-2 antagonism was calculated as the amount of SIV p27 released into the culture supernatant of 293T cells transfected with constructs expressing each of the Nef mutants in the presence of rhesus tetherin relative to the amounts of SIV p27 released in the absence of tetherin.

b Binding to BST-2 was calculated as the relative band intensity of Nef to the band intensity of tetherin in immunoprecipitated samples.

c Steady-state levels of Nef protein in cell lysates were estimated by calculating the relative band intensity of each of the Nef mutant proteins compared to wild-type Nef.

d CD4 levels were calculated as the percentage of CD4 staining (MFI) on cells transfected with each of the indicated Nef-expression constructs relative to CD4 staining (MFI) on cells transfected with an empty vector.

e MHC class I levels were calculated as the percentage of MHC I staining (MFI) on cells transfected with each of the indicated Nef-expression constructs relative to MHC I staining (MFI) on cells transfected an with empty vector.

f GHOST X4/R5 cells were infected with SIV Δ*nef trans*-complemented with the indicated wild-type or mutant Nef proteins, and the relative infectivity was calculated as the frequency of infected cells obtained for each of the mutants relative to wild-type Nef at 48-hours post-inoculation.

Nef mutants that retain wild-type levels of CD4-downregulation, MHC class I-downregulation or infectivity enhancement are indicated in bold and italics. For CD4− and MHC class I-downregulation, one asterisk indicates activity within 5 standard deviations of wild-type Nef and two asterisks indicate activity within 3 standard deviations of wild-type Nef. For infectivity enhancement, two asterisks indicate infectivity 5 standard deviations or more over SIV Δ*nef trans*-complemented with empty vector.

To determine if residues in the flexible loop of SIV Nef are needed for direct binding to rhesus tetherin, purified Nef proteins with deletions in the flexible loop were tested for binding to the cytoplasmic domain of rhesus tetherin by SPR. Recombinant SIV_mac_239 Nef_96–237_ lacking residues 181–200, 181–195, 197–205 and 181–205 were flowed over BIAcore chips coated with a 26 amino acid peptide corresponding to the cytoplasmic domain of rhesus tetherin, as described for Figure 1B. All of the deletion mutants bound to the peptide within a similar range of apparent K_d_ values (Figure 3C; representative raw SPR data is shown in Figure S2A and S2B). The somewhat lower K_d.app_ estimates for three of the deletion mutants (Δ181–200, Δ181–195 and Δ181–205) may be due to technical limitations with testing these mutants at high concentrations as a result of protein aggregation, rather than an actual increase in binding affinity. Some of these mutants also showed lower apparent K_d_ values for binding to a TCRζ chain peptide (Figure S2C–S2E), an interaction that reflects direct binding of the SIV Nef core domain as corroborated by three-dimensional structural data [38]. Hence, these results demonstrate that the flexible loop of Nef is not required for direct binding to rhesus tetherin, implying that surfaces of the core domain are sufficient for the low affinity interaction with the N-terminus of rhesus tetherin observed by SPR.

**Figure 4:**
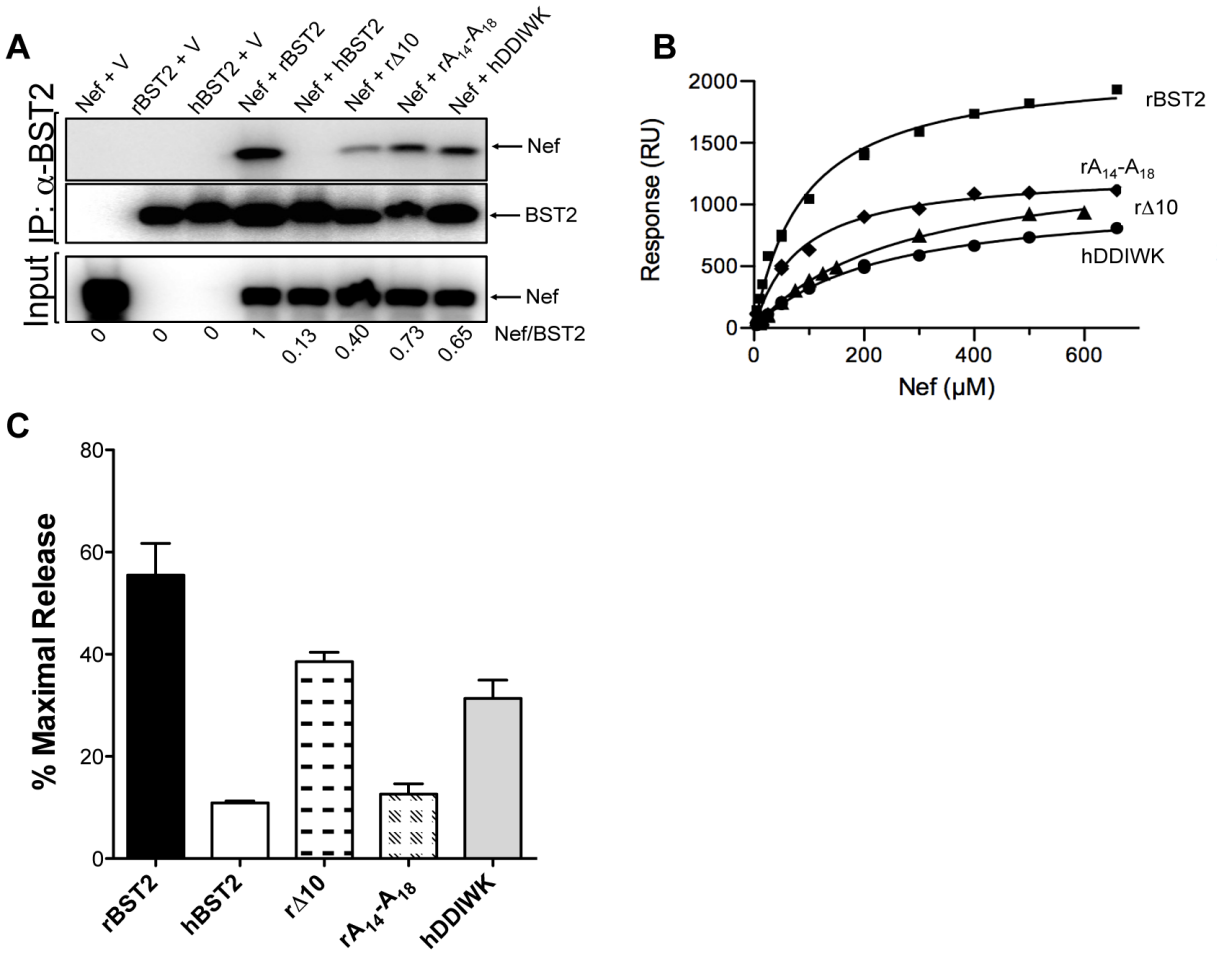
Identification of residues in rhesus tetherin that contribute to interactions with Nef. Mutations in the cytoplasmic domain of rhesus and human tetherin were tested for their effects on binding to Nef by co-immunoprecipitation and SPR assays. (A) Co-immunoprecipitation of Nef in the presence of rhesus, human tetherin and the following tetherin mutants; a rhesus tetherin mutant lacking the first 10 residues of the protein (rΔ10), a rhesus tetherin mutant containing five alanine substitutions at positions 14–18 (rA_14_-A_18_) and a human tetherin mutant containing residues D_14_DIWK_18_ from rhesus tetherin (hDDIWK). The ratios of the band intensities of Nef versus tetherin in the immunoprecipitates are shown beneath each lane. (B) SPR assays were performed as described in Figure 1 to assess binding for Nef_96–237_ and peptides corresponding to the tetherin mutants tested in panel A. (C) Virus release assays were performed to test the susceptibility of each of these tetherin mutants to Nef antagonism. 293T cells were co-transfected with SIV_mac_239 Δ*nef* proviral DNA and constructs coding for rhesus, human tetherin, each tetherin mutant or an empty vector. Constructs coding for Nef were provided *in trans*. Virus release was measured by SIV p27 antigen-capture ELISA and expressed as the percentage of maximal release in the absence of tetherin.

Cytoplasmic domain variants of tetherin were also tested for binding to Nef by co-immunoprecipitation and SPR. Deletion of the first 10 amino acids of rhesus tetherin (rΔ10) significantly reduced, but did not eliminate, binding to Nef in both assays (Figures 4A, 4B and S3B), indicating that although these residues are not essential for binding to Nef, they contribute to the stability of the interaction. Consistent with previous studies mapping the anti-tetherin activity of Nef to a five amino acid sequence (G/D_14_DIWK_18_) that is missing from human tetherin [15], [16], alanine substitutions at positions 14–18 of rhesus tetherin (rA_14_-A_18_) diminished Nef binding, whereas the introduction of these residues into human tetherin partially restored binding (Figures 4A, 4B, S3C and S3D). Thus, although the specificity of tetherin antagonism by Nef is dependent on residues 14–18, and these sequences contribute to a physical interaction with Nef, they are not the sole determinant of Nef binding.

These tetherin variants were also tested in virus release assays to determine how Nef binding relates to susceptibility to antagonism. In accordance with partial binding of Nef to rΔ10 and hDDIWK, restriction of virus release by each of these mutants was partially counteracted by Nef (Figure 4C). However, despite a physical interaction between rA_14_-A_18_ and Nef that was detectable by co-immunoprecipitation and SPR assays, this mutant was resistant to antagonism and restricted virus release to an extent comparable to human tetherin (Figure 4C). Therefore, although a physical interaction may be necessary for tetherin antagonism by Nef, it is not sufficient. This raises the possibility that the anti-tetherin activity of Nef may require the recruitment of one or more additional cellular factors that participate in interactions with the G/D_14_DIWK_18_ sequence.

### Tetherin antagonism is separable from CD4-dowregulation, MHC class I-downregulation and infectivity enhancement

To determine if substitutions that impair tetherin antagonism also disrupt other activities of Nef, the Nef mutants were tested for CD4-downregulation, MHC I-downregulation and infectivity enhancement; three functional activities of Nef that require distinct protein sequences and cellular pathways [39], [40]. CD4− and MHC class I-downregulation assays were performed by electroporating Jurkat cells with bicistronic constructs that express wild-type Nef, or a mutant Nef protein, together with green fluorescent protein (GFP), and comparing the mean fluorescence intensity (MFI) of CD4 and MHC class I staining on the surface of cells expressing Nef to cells transfected with an empty vector (pCGCG) (Figures S4 and S5). Infectivity enhancement was measured by infecting GHOST X4/R5 cells, which express GFP in response to HIV-1 or SIV infection, with SIV_mac_239 Δ*nef trans*-complemented with wild-type Nef or each of the Nef mutants, and measuring the percentage of infected GFP^+^ cells by flow cytometry (Figure S6).

**Figure 5:**
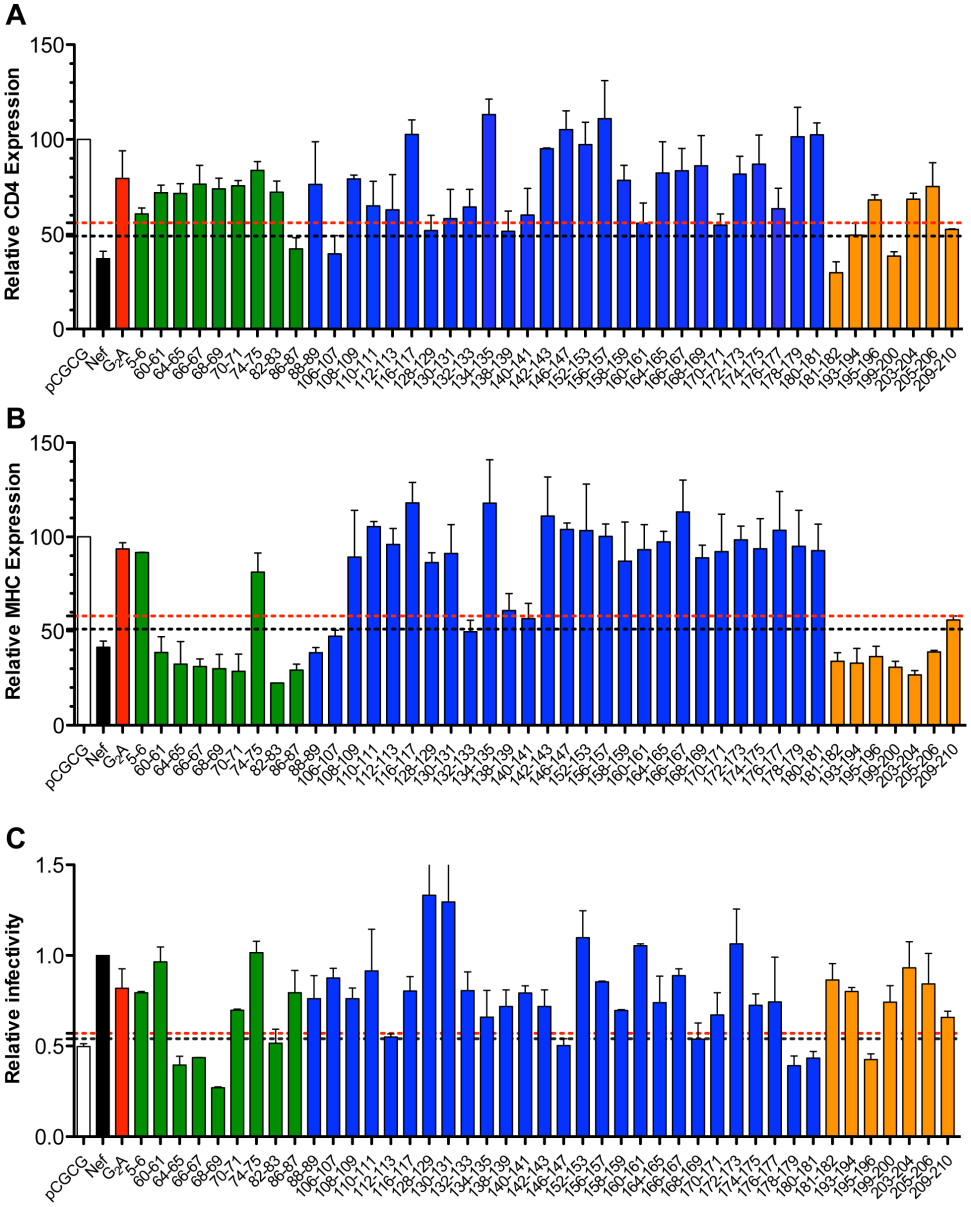
CD4-downregulation, MHC class I-downregulation and infectivity enhancement mediated by SIV Nef mutants with impaired anti-tetherin activity. The surface expression of CD4 (A) and MHC class I (B) was compared on cells expressing each of the SIV Nef mutants. Jurkat cells were electroporated with a bicistronic vector (pCGCG) expressing either wild-type Nef or the indicated Nef mutants together with GFP. Cells were stained with a PerCP-conjugated monoclonal antibody to CD4 and an APC-conjugated monoclonal antibody to HLA class I molecules. Cells were gated on the GFP^+^ cell population and the percentage of CD4 and MHC I on the cell surface was determined relative to cells transfected with empty vector. Error bars indicate the standard deviation of duplicate transfections, and the black and red dotted lines correspond, respectively, to 3 and 5 standard deviations over the activity of wild-type Nef. (C) The Nef mutants with impaired anti-tetherin activity were also tested for infectivity enhancement. Viruses were generated by transient transfection of 293T cells with SIV_mac_239 Δ*nef* proviral DNA and constructs expressing each of the Nef mutants, wild-type Nef or an empty vector. The infectivity of these viruses was then determined on GHOST X4/R5 cells, which express GFP upon SIV infection, 48 hours post-inoculation by flow cytometry. The relative infectivity of SIV Δ*nef trans*-complemented with the Nef mutants was calculated relative to SIV Δ*nef trans*-complemented with wild-type Nef. Virus infectivity in the absence of Nef is indicated by the white bars, and the activities mediated by wild-type Nef and the G_2_A myristoylaton site mutant are indicated by black and red bars, respectively. Error bars indicate the standard deviation of duplicate infections, and the black and red dotted lines correspond, respectively, to 3 and 5 standard deviations over the infectivity of SIV Δ*nef* without *trans*-complementation. Substitutions in the N-terminal domain are indicated by green bars, substitutions in the globular core domain are indicated by blue bars, and substitutions in the flexible loop region are indicated by orange bars.

Of the 43 Nef mutants with impaired anti-tetherin activity, only 5 retained the ability to downregulate CD4 within 3 standard deviations of wild-type Nef (Figure 5A, black dotted line). In contrast, 16 of the mutants retained the ability to downregulate MHC I within 3 standard deviations of wild-type Nef (Figure 5B, black dotted line). Whereas substitutions in the N-terminal domain and flexible loop region, with the exception of substitutions at positions 5–6 and 74–75, had little or no effect on MHC I-downregulation, many of the substitutions in the globular core impaired this activity (Figure 4B and S5). In most cases, the loss of MHC I-downregulation corresponded with a partial decrease in Nef protein levels (Table 1), suggesting that the effects of these mutations were not necessarily specific to this function of Nef. Nevertheless, five Nef mutants with impaired anti-tetherin activity, and no significant effects on protein stability, retained the ability to downregulate both CD4 and MHC I molecules. These included Nef mutants with substitutions at positions 106–107, 181–182, 193–194, 199–200 and 209–210 (Table 1). Therefore, tetherin antagonism is separable from CD4− and MHC class I-downregulation.

The infectivity of SIV Δ*nef trans*-complemented with each of the Nef mutants relative to SIV Δ*nef trans*-complemented with wild-type Nef was also determined to assess the effects of the substitutions on Nef-mediated infectivity enhancement. To control for assay-to-assay variation in the susceptibility of the GHOST X4/R5 cells to infection, the percentage of infected cells obtained for each of the Nef mutants was normalized to the percentage of infected cells obtained for wild-type Nef. Nef mutants were considered to retain the ability to enhance virus infectivity if the relative infectivity was at least 5 standard deviations above the infectivity of SIV Δ*nef trans*-complemented with an empty vector (pCGCG) (Figure 5C). This analysis identified 12 Nef mutants that were impaired for infectivity enhancement (Figure S6A–S6C). In accordance with previous observations, the G_2_A substitution in SIV Nef did not have a significant effect on virus infectivity [41]. Consistent with a study of this function of HIV-1 Nef [40], all of these mutants also lost the ability to bind to dynamin 2 (Dyn2) (Figure S6D). Of these 12 Nef mutants, 10 also exhibited impaired anti-tetherin activity, suggesting that tetherin antagonism and infectivity enhancement may be linked, perhaps by a common dependence on a physical interaction with Dyn2. However, two of the substitutions in the core domain at positions 94–95 and 98–99 that disrupted infectivity enhancement did not significantly affect anti-tetherin activity (Figure 2C and S6B). Moreover, three of the substitutions that disrupted binding to Dyn2 (82–83, 146–147 and 168–169) did not impair binding to rhesus tetherin (Table 2). Thus, Nef appears to use distinct surfaces to bind Dyn2 and tetherin. In addition, since all but 10 of the 43 Nef mutants lacking anti-tetherin activity retained the ability to enhance infectivity, including the 5 mutants that retained both CD4− and MHC class I-downregulation, infectivity enhancement is independent of tetherin antagonism (Figure 5C and Table 1).

**Table 2:** Comparison of binding to Dyn2 and tetherin for Nef substitutions with impaired infectivity enhancement.

	Infectivity enhancement^a^	Dyn2 binding^b^	BST2 binding^c^
**Nef**	1	1.1	1
**G_2_A**	0.81	0.17	0.16
**64–65**	0.39	0.35	0.54
**66–67**	0.43	0.46	0.18
**68–69**	0.27	0.50	0.14
**82–83**	0.51	0.26	1.38
**94–95**	0.58	0.17	NT
**98–99**	0.49	0.50	NT
**112–113**	0.59	0.18	0.35
**146–147**	0.50	0.35	1.56
**168–169**	0.54	0.52	2.1
**195–196**	0.42	0.22	0.31

a Infectivity enhancement was calculated as the relative infectivity of Δ*nef*-viruses *trans*-complemented with these Nef mutants to Δ*nef*-viruses *trans*-complemented with wild-type Nef.

b Binding to dynamin 2 was calculated as the relative band intensity of Nef to the band intensity of dynamin 2 in immunoprecipitated samples.

c Binding to tetherin was calculated as the relative band intensity of Nef to the band intensity of tetherin in immunoprecipitated samples.

### Mutations in the flexible loop of SIV Nef impair tetherin downregulation and AP-2 binding

**Figure 6:**
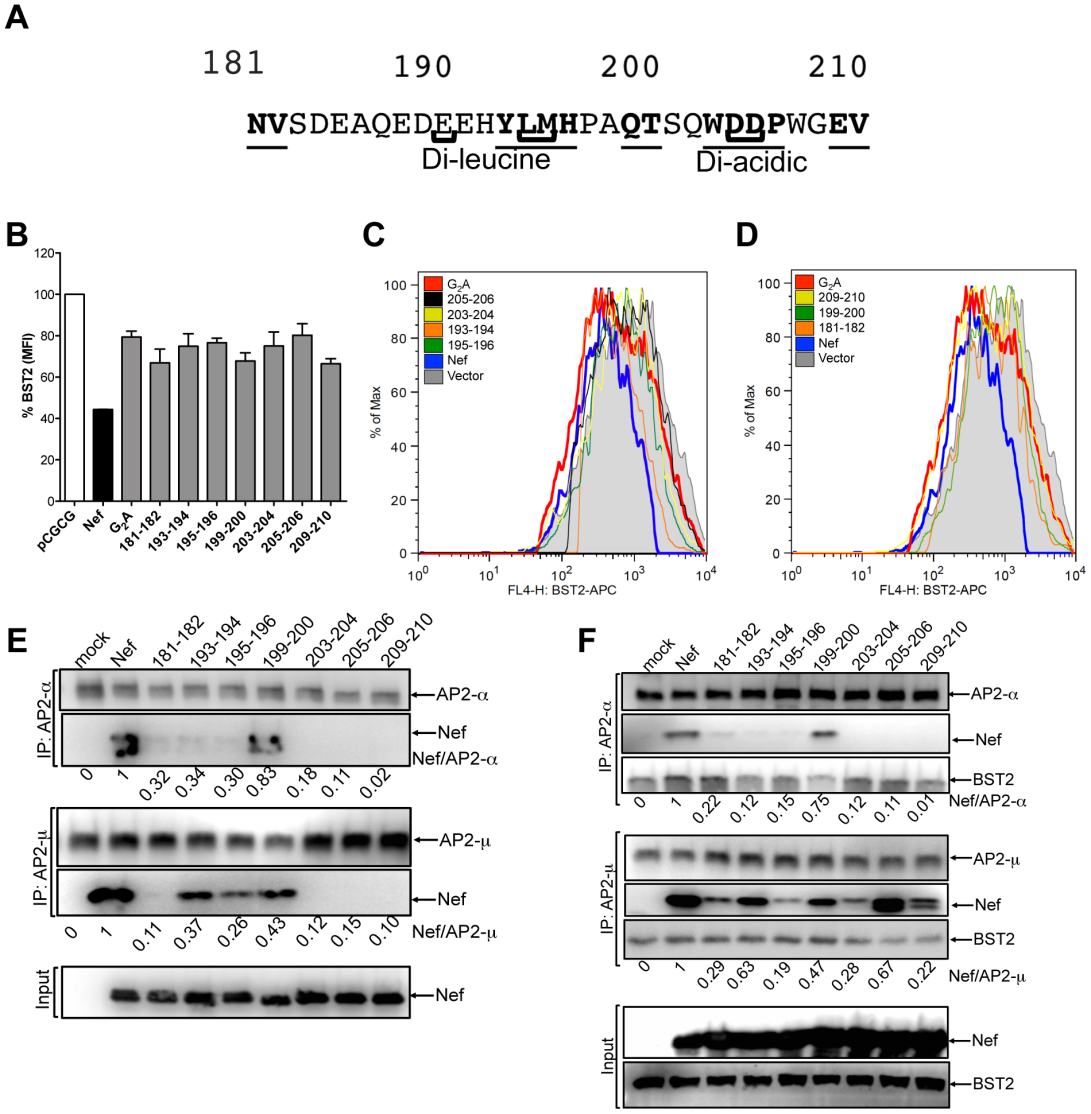
Contribution of flexible loop residues to Nef-mediated antagonism of tetherin and binding to AP-2. (A) Predicted amino acid sequence of the flexible loop region of the SIV_mac_239 Nef protein. Substitutions at positions that impair anti-tetherin activity are underlined and in bold. The di-leucine and di-acidic motifs known to interact with AP-2 are indicated in brackets. (B) Tetherin downregulation by each of the flexible loop mutants was assessed by transfecting 293T cells that constitutively express HA-tagged rhesus tetherin with constructs expressing the indicated Nef mutants, and comparing the cell surface expression of tetherin to cells transfected with the empty vector (pCGCG). The percentage of tetherin expressed on the cell surface was calculated by dividing the MFI of tetherin (HA) staining on cells expressing Nef by the MFI of tetherin on cells transfected with the empty vector. (C and D) Histogram plots showing the surface expression of tetherin for cells expressing Nef mutants with substitutions in known AP-2 binding sites (C) and at sites flanking these residues (D). (E and F) Nef mutants with substitutions in the flexible loop were tested for binding to the α2 and μ2 subunits of AP-2 by co-immunoprecipitation. Parental 293T cells (E) or 293T cells that constitutively express HA-tagged rhesus tetherin (F) were transfected with constructs expressing the indicated Nef mutants, and cell lysates were immunoprecipitated with monoclonal antibodies to the α2 and μ2 subunits of AP-2. Western blots of immunoprecipitates and cell lysates were probed with antibodies to α2, μ2, Nef and tetherin. The ratios of the band intensities of Nef to α2 and Nef to μ2 in the immunoprecipitated samples are shown beneath each lane.

AP-2 binds to a pair of conserved motifs in the flexible loop of Nef that are necessary for tetherin antagonism; a di-leucine motif and a di-acidic motif, corresponding to residues E_191_XXXLM_195_ and D_204_D_205_ of SIV_mac_239 Nef, respectively (Figure 6A) [37], [42], [43]. Consistent with previous observations [37], substitutions of residues within either of these motifs (positions 193–194, 195–196, 203–204, 205–206) impaired tetherin downregulation by Nef (Figure 6B and 6C). In addition, substitutions at positions 181–182, 199–200 and 209–210, not previously identified as AP-2 binding sites or known to be involved in the anti-tetherin activity of Nef, also impaired tetherin downregulation (Figure 6B and 6D). These results confirm the role of the di-leucine and di-acidic motifs and identify additional sequences in the flexible loop of SIV Nef required for tetherin downmodulation.

Since AP-2 binds to both Nef and tetherin [42], [43], [44], the flexible loop mutants were also tested for their ability to interact with the α-adaptin (α2) and μ2 subunits of AP-2 by co-immunoprecipitation. Endogenous α2 and μ2 were immunoprecipitated in parallel from lysates of parental 293T cells (Figure 6E), or 293T cells that constitutively express HA-tagged rhesus tetherin (Figure 6F), following transfection with Nef expression constructs. Immunoprecipitates were separated by electrophoresis and western blots were probed with antibodies to Nef, α2, μ2 and tetherin. Wild-type Nef co-immunoprecipitated with α2 and μ2, both in the absence and in the presence of tetherin (Figures 6E and 6F). As expected, substitutions in the di-leucine and di-acidic motifs of Nef (193–194, 195–196, 203–204, 205–206) greatly diminished binding to both subunits (Figures 6E and 6F). Substitutions at positions flanking these motifs (181–182 and 209–210) also disrupted binding to α2 and to μ2 (Figures 6E and 6F). Whereas in the absence of tetherin, substitutions at positions 181–182, 203–204, 205–206 and 209–210 eliminated Nef binding to μ2, and substitutions at positions 193–194, 195–196, and 199–200 reduced Nef binding to μ2, (Figure 6E), the binding of these Nef mutants to μ2 was partially restored in the presence of tetherin (Figure 6F). These results suggest that the loss of anti-tetherin activity for each of the flexible loop mutants reflects a deficit in Nef binding to AP-2, and raises the possibility that AP-2 may form a multimeric complex with both Nef and tetherin that stabilizes an otherwise low affinity direct interaction between these two proteins.

### Tetherin antagonism by Nef is dependent on dynamin 2 and clathrin

**Figure 7:**
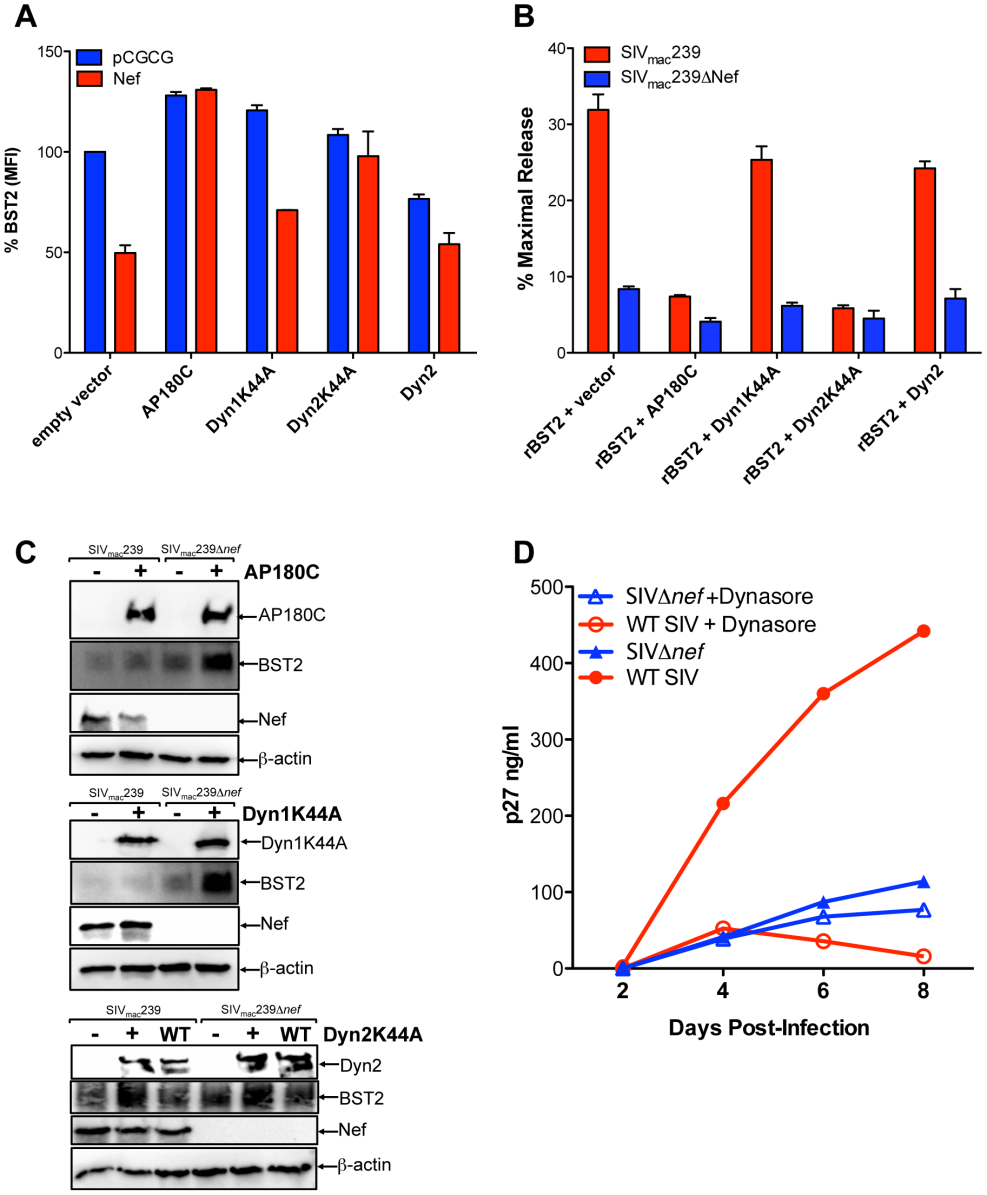
Tetherin antagonism by Nef depends on clathrin and dynamin 2. (A) Dominant-negative mutants of AP180 and Dyn2 were tested for disruption of Nef-mediated tetherin downregulation. 293T cells expressing HA-tagged rhesus tetherin were transfected with constructs expressing Nef, or empty vector, and constructs expressing AP180C, Dyn1K44A, Dyn2K44A and Dyn2. Surface expression of tetherin was measured by HA staining, and the percentage of tetherin expressed on the cell surface was determined by dividing the MFI of tetherin on cells expressing the dominant-negative mutants by the MFI of tetherin on cells transfected with empty vectors. (B) The dominant-negative mutants of AP180 and Dyn2 were also tested for their effects on Nef-mediated virus release. 293T cells were transfected with SIV_mac_239 or SIV_mac_239 Δ*nef* proviral DNA, and constructs expressing rhesus tetherin, and either AP180C, Dyn1K44A, Dyn2K44A, Dyn2 or empty vector. Virus release was measured by SIV p27 antigen-capture ELISA and expressed as the percentage of maximal release in the absence of tetherin. (C) Protein expression for Nef, tetherin, AP180C, Dyn1K44A, Dyn2K44A and Dyn2 was verified by western blot analyses using endogenous β-actin as a loading control. (D) Replication of wild-type SIV (WT SIV) versus SIV Δ*nef* in the presence and absence of Dynasore. 221 T cells were infected with 20 ng p27 of wild-type SIV or SIV Δ*nef*. Twenty-four hours post-infection, cells were treated with IFNα (100 U). Eight hours later, Dynasore (20 µM) was added to one of the cultures. Supernatants were collected at the indicated time points and virus replication was determined by SIV p27 antigen-capture ELISA.

Since the activities of HIV-1 Vpu and HIV-2 Env in downregulating tetherin and facilitating virus release were recently shown to be dependent on AP180, a component of the clathrin assembly complex [30], and dynamin 2 (Dyn2), an ubiquitously expressed GTPase required for the scission of vesicular membranes [45], we asked whether tetherin antagonism by Nef also requires AP180 and Dyn2. The effects of dominant-negative mutants of Dyn2 (Dyn2K44A) and AP180 (AP180C) on the surface expression of tetherin, and on virus release, were therefore tested in 293T cells expressing HA-tagged rhesus macaque tetherin. As a control, we also included the dominant-negative mutant of dynamin 1 (Dyn1K44A), which is exclusively expressed in neurons [46]. Changes in tetherin expression on the cell surface were assessed by flow cytometry after co-transfection with constructs expressing Dyn2K44A, Dyn2, Dyn1K44A or AP180C, with or without Nef (Figure 7A). In the absence of Nef, Dyn1K44A, Dyn2K44A and AP180C all slightly increased surface levels of tetherin, whereas wild-type Dyn2 decreased surface levels of tetherin (Figure 7A), which may reflect a role for dynamin and clathrin in the constitutive endocytosis of tetherin [44]. In the presence of Nef, the effects of the dominant-negative mutants were more pronounced. Whereas SIV Nef reduced the surface expression of rhesus tetherin by 2- to 3-fold, as previously reported [15], this effect was almost completely reversed by AP180C and Dyn2K44A (Figure 7A). Although Dyn1K44A also increased the overall levels of tetherin at the cell surface, as shown in transfections with the empty vector, Nef was still able to downregulate tetherin in the presence of Dyn1K44A (Figure 7A). Likewise, in the presence of wild-type Dyn2, Nef further decreased the surface levels of tetherin. These results demonstrate that the downregulation of tetherin by Nef is dependent, at least in part, on clathrin-mediated endocytosis.

To further confirm that Dyn2 and clathrin are required for tetherin antagonism by Nef, virus release for wild-type SIV versus SIV Δ*nef* was compared in the presence and absence of each of the dominant-negative mutants. 293T cells were co-transfected with proviral DNA for SIV_mac_239 or SIV_mac_239 Δ*nef*, together with a construct expressing rhesus macaque tetherin and expression constructs for AP180C, Dyn1K44A, Dyn2K44A or Dyn2, and the accumulation of virus particles in the cell culture supernatant was measured by SIV p27 antigen-capture ELISA. Whereas AP180C and Dyn2K44A completely abrogated the resistance of wild-type SIV to rhesus tetherin, as indicated by comparable levels of virus release for SIV_mac_239 and SIV_mac_239 Δ*nef*, virus release for wild-type SIV was not significantly affected by Dyn1K44A or Dyn2 (Figure 7B). Western blot analyses of cell lysates confirmed protein expression for tetherin, Nef and the dominant-negative mutants (Figure 7C). None of the dominant-negative mutants inhibited tetherin expression. On the contrary, increased steady-state levels were observed in the presence of AP180C, Dyn1K44A and Dyn2K44A (Figure 7C), consistent with the modest increase in cell surface expression of tetherin in the absence of Nef (Figure 7A).

A role for Dyn2 in the anti-tetherin activity of Nef was further investigated by comparing virus replication of wild-type SIV and *nef*-deleted SIV with or without Dynasore, a chemical inhibitor of dynamin. A *Herpesvirus saimiri*-immortalized rhesus macaque CD4^+^ T cell line [47], was infected with SIV_mac_239 and SIV_mac_239 Δ*nef*, treated with IFNα to upregulate tetherin, and maintained in medium with or without Dynasore. While Dynasore had little effect on the replication of SIV Δ*nef*, which was suppressed relative to wild-type SIV by the IFNα-induced upregulation of tetherin, Dynasore significantly reduced wild-type SIV replication (Figure 7D). Indeed, wild-type SIV replication in the presence of Dynasore was comparable to SIV Δ*nef* replication, suggesting that this compound fully negated the resistance provided by Nef to the antiviral effects of tetherin. However, since Dyn2 is also required for infectivity enhancement by Nef [40], these results may reflect an additional effect of Dynasore on viral infectivity.

### Nef promotes the internalization of tetherin in SIV-infected cells

**Figure 8:**
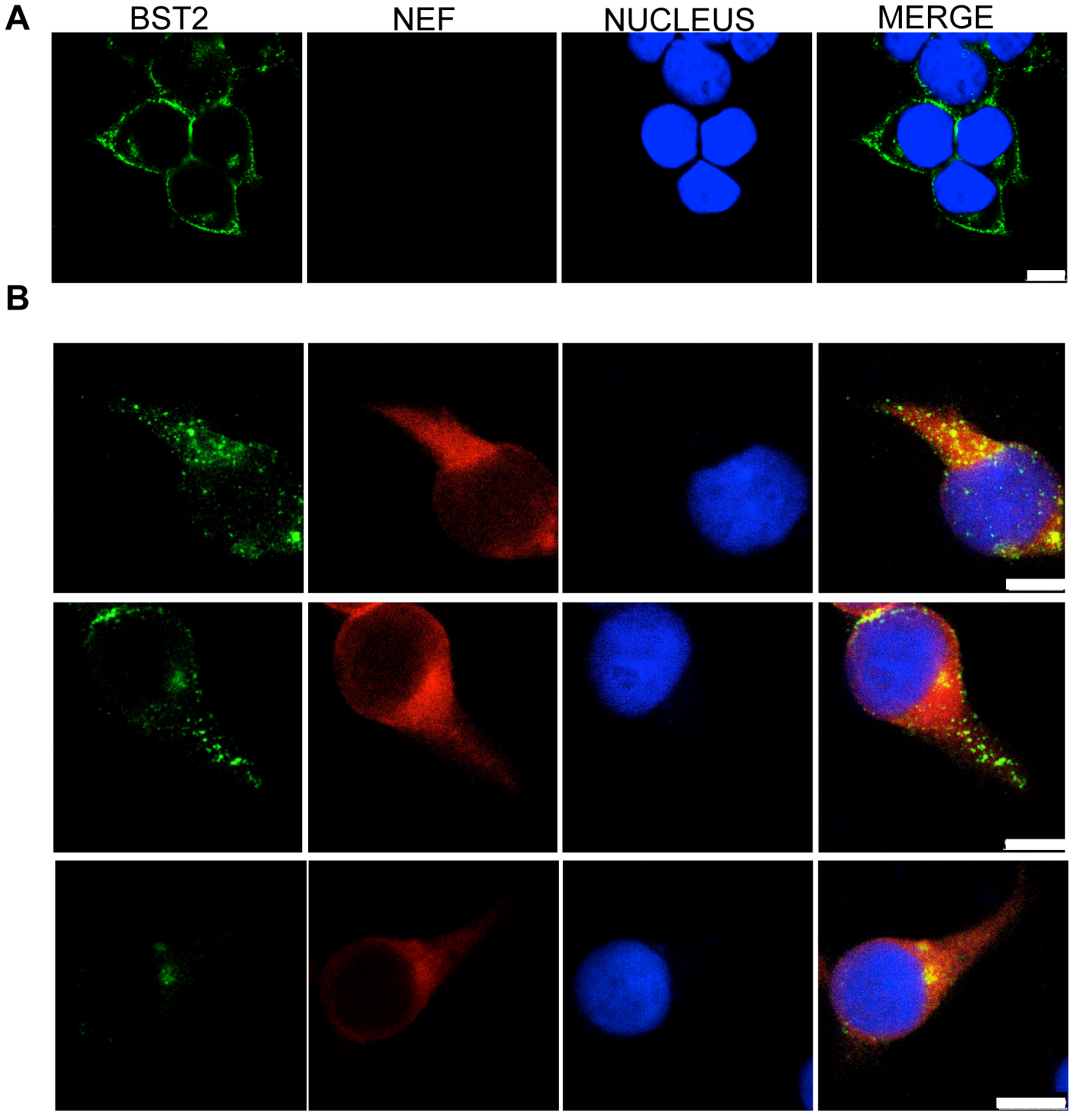
Distribution of Nef and tetherin in SIV-infected cells. 293T cells expressing HA-tagged rhesus tetherin were infected with VSV-G pseudotyped SIV_mac_239 Δ*env* and stained for tetherin (green), Nef (red) and the cell nucleus (blue). Tetherin expression in uninfected cells (A), and the localization of Nef and tetherin in SIV-infected cells (B). The white scale bar indicates 10 µm.

**Figure 9:**
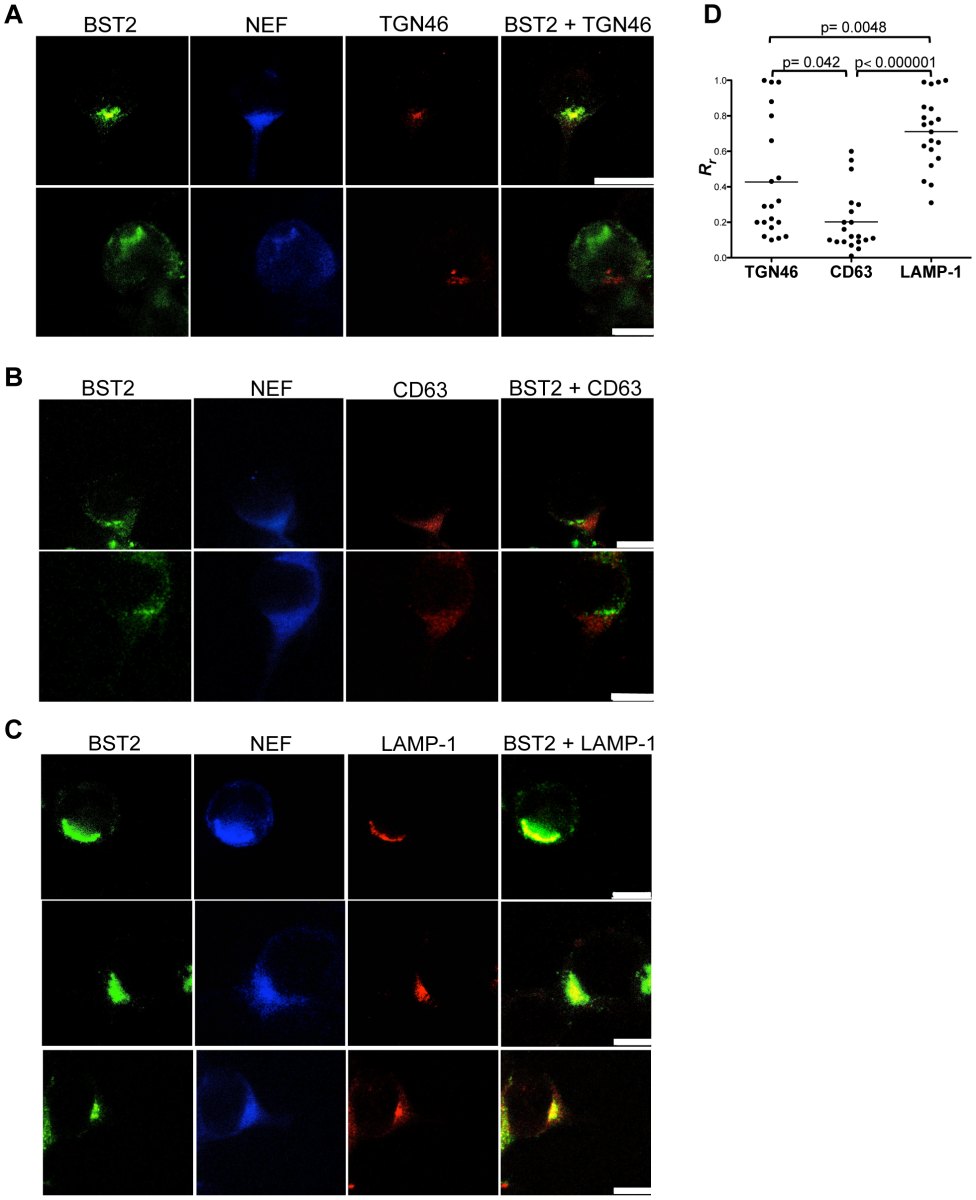
Subcellular distribution of tetherin in SIV-infected cells. 293T cells expressing HA-tagged rhesus macaque tetherin were infected with VSV-G pseudotyped SIV_mac_239 Δ*env* and stained for tetherin (green), Nef (blue) and either TGN46 (red) (A), CD63 (red) (B) or LAMP-1 (red) (C). The white scale bar indicates 10 µm. (D) The extent of co-localization between tetherin and each of the intracellular markers was estimated by calculation of the Pearson's correlation coefficients for images of twenty randomly selected SIV-infected cells.

Changes in the subcellular distribution of tetherin in the presence of Nef were examined in uninfected and SIV-infected cells by confocal microscopy. 293T cells expressing HA-tagged rhesus tetherin were infected with VSV-G-pseudotyped SIV_mac_239 Δ*env* and stained for Nef and tetherin. In uninfected cells, tetherin was observed at the plasma membrane and within the *trans*-Golgi network (Figure 8A and Figure S7), as previously reported [48]. However, in SIV-infected cells, the overwhelming majority of tetherin was observed within intracellular compartments (Figure 8B). To better define the subcellular distribution of tetherin, cells were stained for markers of the *trans*-Golgi network (TGN46), endosomes (CD63) and lysosomes (LAMP-1). In some cells, tetherin co-localized with TGN46 (Figure 9A), but did not appear to co-localize with CD63 (Figure 9B). These results suggest that Nef may partially retain tetherin within the *trans*-Golgi network with little or no sequestration in endosomes. However, in the majority of the SIV-infected cells, tetherin was found to co-localize with LAMP-1 (Figure 9C), but not in uninfected cells (Figure S7). These observations were supported by quantifying the extent of tetherin co-localization with TGN46, CD63 and LAMP-1 by calculating the Pearson's correlation coefficients for these markers in twenty SIV-infected cells. Although the distribution of cells exhibiting co-localization of tetherin with TGN46 was heterogeneous (Figure 9D), it was higher than the extent of co-localization with CD63 (P = 0.042). In the case of LAMP-1, the extent of co-localization with tetherin was significantly higher than for either TGN46 (P = 0.0048) or CD63 (P<0.000001). Therefore, similar to the effects of Nef on CD4 and MHC class I trafficking [49], Nef appears to direct tetherin to lysosomes.

## Discussion

The primate lentiviruses have evolved to use at least three different proteins to counteract tetherin; Nef, Vpu and Env [5], [13], [15], [16], [32], [36], [50]. Although a number of studies have addressed the mechanisms of tetherin antagonism by HIV-1 Vpu and HIV-2 Env [4], [5], [13], [18], [23], [27], [29], [30], [31], [51], relatively little is known about the mechanism of tetherin antagonism by Nef–the viral gene product used by most SIVs to counteract the tetherin proteins of their respective hosts. In accordance with the species-dependent activity of Nef in opposing restriction by tetherin [15], [16], we show for the first time that Nef selectively binds to rhesus macaque tetherin, but not to human tetherin. We identify residues in the N-terminus, globular core and flexible loop of Nef that are required for anti-tetherin activity, and demonstrate that, despite substantial overlap with sequences required for CD4 downregulation, tetherin antagonism is genetically separable from this activity, as well as from other Nef functions including MHC class I downregulation and infectivity enhancement. We also show that dominant-negative mutants of AP180 and Dyn2 impair tetherin antagonism by Nef, indicating that this activity is dependent, at least in part, on clathrin-mediated endocytosis.

Co-immunoprecipitation and surface plasmon resonance assays revealed a selective physical interaction between SIV Nef and rhesus tetherin. The specificity of this interaction is determined by binding of the core domain of Nef to the cytoplasmic domain of tetherin, since a truncated form of the SIV_mac_239 Nef protein, containing the globular core of the protein, was sufficient for binding to a peptide corresponding to the cytoplasmic domain of rhesus tetherin. However, the affinity of this interaction was low (K_d_∼400 µM), suggesting that additional Nef sequences, and perhaps one or more cellular co-factors, contribute to the stability of this interaction in virus-infected cells. In support of this, an analysis of alanine-scanning substitutions identified sequences in the N-terminal, globular core and flexible loop domains of Nef that participate in binding to rhesus tetherin. Although the N-terminal domain and flexible loop were dispensable for binding by SPR, these sequences were required to detect an interaction by co-immunoprecipitation. The contribution of the N-terminus of Nef to interactions with tetherin may reflect an indirect effect on membrane association, since the targeting of Nef to cellular membranes is dependent on the myristyolation of a glycine residue at position 2, and structural studies suggest that the N-terminus of Nef is disordered in the absence of phospholipids [52], [53], [54]. Substitutions at positions 116–117, 174–175 in the globular core domain, and positions 181–182, 193–194, 195–196 and 199–200 in the flexible loop region also reduced binding to rhesus tetherin. Since the flexible loop contains a di-leucine and a di-acidic motif (E_190_xxxLM and D_204_D) required for binding to the AP-2 subunits (α2-σ2 and μ2, respectively) [42], [55], [56], and substitutions in these sites disrupt tetherin antagonism [37], it is conceivable that AP-2 stabilizes the binding of Nef to tetherin. Indeed, Nef was recently shown to form a trimolecular complex with the μ1 subunit of AP-1 to stabilize an otherwise low affinity bimolecular interaction with the cytoplasmic tail of MHC class I molecules [57], [58]. In support of a possible trimeric complex with AP-2, Nef and rhesus tetherin both co-immunoprecipitated with the μ2 and α2 subunits of AP-2 [24], [44]. Taken together, these results suggest a model in which the specificity of SIV Nef for rhesus tetherin is driven by a direct physical interaction between the core domain of Nef and the N-terminus of tetherin, which is stabilized by residues in the N-terminal domain and flexible loop region, either through direct contacts or indirect effects on membrane association and/or the recruitment of additional cellular co-factor(s).

A systematic analysis of alanine-scanning substitutions throughout the SIV_mac_239 Nef protein identified a total of 43 mutations that impaired anti-tetherin activity. Substitutions in the C-terminal domain were not tested, since deletion of these sequences did not affect tetherin antagonism. Most of the mutations that disrupted the anti-tetherin activity of Nef also disrupted CD4-downregulation, MHC I-downregulation or infectivity enhancement. In some cases, both CD4− and MHC I-downregulation were lost due to effects on the association of Nef with cellular membranes, such as the G_2_A mutation and probably also the adjacent substitutions at positions 5–6. In other cases, these activities were lost due to a decrease in Nef expression or stability. While this was most evident for the changes at positions 178–179 and 180–181, some of the substitutions in the globular core domain also had partial effects on steady-state levels of Nef that may account for their reduced activity in CD4 and MHC class I downregulation assays. Although there was substantial overlap with sequences required for CD4-downregulation, five mutations were identified that disrupted the anti-tetherin activity of Nef, while retaining nearly wild-type levels of CD4-downregulation, as well as MHC class I-downregulation and infectivity enhancement. These mutations included alanine substitutions at positions 106–107 in the core domain and at positions 181–182, 193–194, 199–200 and 209–210 in the flexible loop region. Thus, tetherin antagonism by Nef is genetically separable from other functional activities of the protein.

In addition to the sequences identified by Zhang et al. [37], we identified residues in the flexible loop region outside of the known AP-2 binding sites that separate tetherin antagonism from CD4-downregulation. Substitutions at positions 181–182 and 199–200 (residues N_181_V_182_ and Q_199_T_200_ in SIV_mac_239 Nef) specifically impaired the anti-tetherin activity of Nef without affecting CD4-downregulation. These residues are well conserved among Nef alleles of SIV_smm/mac_ and HIV-2 isolates, with identities of 61.1% for N_181_, 75% for V_182_, 94.4% for Q_199_ and 84.7% for T_200_ (Los Alamos database; http://www.hiv.lanl.gov/content/index). Co-immunoprecipitation assays further demonstrated that these residues contribute to AP-2 binding. Our mutational analysis also identified residues in the N-terminal and globular core domains of Nef that are important for tetherin antagonism. Thus, our results reveal that the anti-tetherin activity of Nef is dependent on complex interactions involving multiple residues in the N-terminus, globular core and the flexible loop regions of the protein.

Nef is a multifunctional accessory protein that interacts with a number of different cellular factors to modulate cellular trafficking [55]. Nef reroutes MHC I molecules from the *trans*-Golgi network to lysosomes via AP-1 and promotes the internalization and lysosomal degradation of CD4 via AP-2 [55], [59], [60], [61]. Nef also enhances virus infectivity by an undefined mechanism that depends on a physical interaction with Dyn2 [40]. We previously demonstrated that Nef downmodulates tetherin from the surface of SIV-infected and transfected cells [15], [36], and this activity was later shown to occur by an AP-2-dependent pathway [37]. Our experiments with dominant-negative mutants of AP180 and Dyn2 confirm that the internalization of tetherin by Nef, and the capacity of Nef to rescue virus release in the presence of tetherin, depends, at least in part, on clathrin-mediated endocytosis. A role for Dyn2 was further demonstrated by showing that Dynasore, a chemical inhibitor of dynamin, suppressed wild-type SIV replication to an extent comparable to *nef*-deleted SIV under conditions of interferon-induced upregulation of tetherin. Since Dyn2 is also required for Nef-mediated infectivity enhancement, the inhibition of virus replication by Dynasore may reflect an additional effect of this compound on virus infectivity. Although tetherin antagonism and infectivity enhancement are genetically separable, 10 of the 12 Nef mutants that lost the ability to enhance virus infectivity, and to bind to Dyn2, also lost the ability to counteract tetherin. The concordance of these activities suggests that a physical interaction with Dyn2 may be necessary for both Nef functions. However, two of the mutations in the globular core disrupted infectivity enhancement and binding to Dyn2 without impairing tetherin antagonism. Moreover, three of the mutants with impaired infectivity enhancement and binding to Dyn2 (mutants 82–83, 146–147 and 168–169) did not lose binding to tetherin, suggesting that Nef uses distinct protein surfaces to bind to Dyn2 and to tetherin. Therefore, unlike infectivity enhancement, the anti-tetherin activity of Nef does not depend on a physical interaction with Dyn2.

Consistent with previous studies demonstrating the downmodulation of rhesus tetherin by Nef [15], [36], [37], SIV infection resulted in a striking redistribution of tetherin from the plasma membrane to compartments within the cell. An analysis of the distribution of tetherin in SIV-infected cells revealed co-localization with TGN46 and LAMP-1, but not with CD63, suggesting that in the presence of Nef, tetherin accumulates in the *trans*-Golgi network and in lysosomes. Localization of tetherin to the *trans*-Golgi network in uninfected cells has previously been reported [48]. Thus, the contribution of Nef to directing tetherin to that compartment is unclear. The trafficking of tetherin to lysosomes raises the possibility that, similar to the effect of HIV-1 Nef on CD4 and MHC class I molecules [49], SIV Nef may direct rhesus tetherin for lysosomal degradation.

In summary, we show that the mechanism of tetherin antagonism by SIV Nef; (1) involves a direct physical interaction between the core domain of Nef and the cytoplasmic domain of rhesus tetherin, (2) requires sequences throughout the N-terminal, globular core and flexible loop domains, yet is genetically separable from other functional activities of Nef, and (3) depends, at least in part, on clathrin-mediated endocytosis. These results begin to reveal the molecular interactions and cellular pathways by which the majority of the primate lentiviruses counteract the tetherin proteins of their non-human primate hosts.

## Materials and Methods

### Plasmid DNA constructs

#### (a) *BST 2* expression constructs

Human *BST-2* (*hBST-2*) and rhesus macaque *BST-2* allele *rBST-2.1* were cloned as previously described [15], [36].

#### (b) SIV proviral clones

Full-length proviral DNA clones for wild-type SIV, SIV Δ*env* and SIV Δ*nef* were constructed from the following clones based on SIV_mac_239; p239SpSp5', pSP72-239-3' and pSP72-239-3'Δ*nef* [62], [63].

#### (c) Nef expression constructs

SIV Nef was cloned into the expression vector pCGCG-239-Nef as previously described [15], [36]. Mutations in Nef were introduced by Quickchange site-directed mutagenesis according to the manufacturer guidelines (Stratagene, La Jolla, CA).

#### (d) Dominant-negative mutants

The dominant negative mutant AP180C and Dyn1K44A were kindly provided by Dr. Min Dong (New England Primate Research Center, Southborough, MA). AP180C was flag-tagged and cloned into pCMV, whereas Dyn1K44A was HA-tagged and cloned into pCDNA. The dominant-negative mutant Dyn2K44A, as well as wild-type Dyn2, was kindly provided by Dr. John Guatelli. These constructs were cloned into pEGFP N1.

### Virus release assays

293T cells were co-transfected with wild-type or *nef*-deleted SIV proviral DNA (100 ng) and pcDNA3-tetherin or pcDNA3-tetherin mutants (50 ng). Differences in the amount of plasmid DNA in each transfection were offset by the addition of empty pcDNA3 vector (50 ng). Either pCGCG, pCGCG-Nef or pCGCG-Nef mutants (100 ng each) were provided *in trans* to assess the ability of the Nef mutants to rescue virus release. All transfections were performed in duplicate in 24-well plates seeded the day before at 5×10^4^ cells per well, using GenJet Lipid Transfection Reagents (SignaGen Laboratories, Gaithersburg, MD). Forty-eight hours post-transfection, the amount of virus released into the cell culture supernatant was measured by SIV p27 antigen-capture ELISA (Advanced Bioscience Laboratories, Inc., Kensington, MD), and virus release was expressed as the percentage of maximal particle release in the absence of tetherin, as previously described [15], [36].

### Western blots

Forty-eight hours post-transfection, 293T cell lysates were prepared by harvesting in 2× SDS sample buffer. Virions were recovered from the cell culture supernatant by centrifugation at 13,000 rpm for 2 hours at 4°C, and resuspended in 2× SDS sample buffer. Samples were boiled for 5 minutes, and separated by electrophoresis on 12% SDS-polyacrylamide gels and transferred to polyvinylidine fluoride (PVDF) membranes using a Trans-Blot SD transfer cell (BioRad, Hercules, CA). The membranes were then blocked with 5% non-fat dry-milk in PBS containing 0.05% Tween-20 for 1 hour, and probed overnight at 4°C with one of the following primary antibodies. Tetherin/BST-2 was detected with a mouse polyclonal antibody (abcam cat #ab88523, Cambridge, MA) at a dilution of 1∶500. The SIV Gag proteins p27 and p55 were detected with the mouse monoclonal antibody 183-H12-5C (AIDS Research and Reference Reagent Program, Division of AIDS, NIAID, NIH) at a dilution of 1∶1000. SIV Nef was detected using the mouse monoclonal antibody 17.2 (AIDS Research and Reference Reagent Program, Division of AIDS, NIAID, NIH) at a dilution of 1∶1000. Endogenous β-actin was detected with the monoclonal antibody C4 (Chemicon, Billerica, MA) at a dilution of 1∶1000. HA-tagged Dyn1K44A was detected with the HA-specific mouse monoclonal antibody HA.11 (Covance, Princeton, NJ) at a dilution of 1∶1000. The GFP fusion proteins Dyn2 and Dyn2K44A were detected using an anti-GFP mouse monoclonal antibody (Sigma-Aldrich, St Louis, MO) at a dilution of 1∶1000. The dominant-negative mutant AP180C was detected with a mouse monoclonal FLAG-specific antibody (Sigma-Aldrich, St Louis, MO) at a dilution of 1∶1000. After rinsing the PVDF membranes three times for 10 minutes in PBS 0.05% Tween-20, the blots were probed with an HRP-conjugated goat anti-mouse secondary antibody (Pierce, Rockford, IL) at a dilution of 1∶2000 for 1 hour at room temperature. The blots were then rinsed three more times in PBS 0.05% Tween-20, treated with SuperSignal West Femto Maximum Sensitivity substrate (Pierce, Rockford, IL), and imaged using a Fujifilm Image Reader LAS 3000 (Fujifilm Photo Film Co., Japan).

### Co-immunoprecipitation assays

293T cells (6×10^5^ cells) were co-transfected with constructs expressing wild-type and mutant forms Nef (2 µg) along with rhesus tetherin, human tetherin, tetherin mutants, Dyn2-GFP or empty vector (pCDNA3) (2 µg). Twenty-four hours later, cells were lysed with 400 µl of Lysis buffer (Thermo Scientific, Rockford, IL) and incubated on ice for 30 minutes. Lysates were transferred to a 1.5 ml tube and insoluble cell debris was removed by centrifugation at 3,000 rpm. Cell lysate (200 µl) was set aside to confirm tetherin and Nef expression by western blot analysis, and the rest of the sample (200 µl) was used for immunoprecipitation. Samples for immunoprecipitation were incubated on a rotating platform for 1 hour at 4°C with 1 µg of the anti-tetherin mouse monoclonal antibody 3H4 (Sigma-Aldrich, St Louis, MO). Protein A sepharose beads or Protein A sepharose magnetic beads (50 µl) (GE Healthcare, Piscataway, NJ) were then added, and the incubation was continued overnight at 4°C. The beads were washed ten times in Lysis buffer (500 µl) and boiled in 2× SDS sample buffer. Denatured proteins were separated on 12% SDS-polyacrylamide gels and transferred to PVDF membranes. The blots were probed with the monoclonal antibody 17.2 to detect Nef, a mouse monoclonal to detect GFP (Sigma-Aldrich, St Louis, MO), a monoclonal antibody to detect the α2 subunit of AP-2 (Sigma-Aldrich, St Louis, MO), a rabbit monoclonal to detect the μ2 subunit of AP-2 (abcam, Cambridge, MA) at a dilution of 1∶1000, or a polyclonal antibody against tetherin (abcam, Cambridge, MA) at a dilution of 1∶500. Membranes were next probed with an HRP-conjugated goat anti-mouse antibody (Pierce, Rockford, IL), a goat anti-mouse heavy chain specific antibody (abcam, Cambridge, MA), or goat anti-rabbit secondary antibody (abcam, Cambridge, MA), developed in SuperSignal West Femto Maximum Sensitivity substrate and imaged using a Fujifilm Image Reader LAS 3000 as described above. Quantification of the association between Nef and rhesus tetherin, Nef and AP-2 or Nef and Dyn2 was performed by determining the band density from western blots using ImageJ software (Rasband, W.S., Image, US. NIH, Bethesda, MD, http://rsb.info.nih.gov/ij, 1997–2008).

### Tetherin cytosolic domain peptides

Synthetic peptides (21^st^ Century Biochemicals) corresponding to the cytoplasmic domain of rhesus macaque (MAPILYDYRKMPMDDIWKEDGDKR**C**K) and human (MASTSYDY*S*RVPMEDGDKR**C**K) tetherin were biotinylated via stable thioester bonds at conserved cysteine residues (underlined in sequences shown above). In human tetherin cysteine at position 9 was replaced by serine (italics) in order to avoid multiple labeling. Additional tetherin peptides (rA_14_-A_18_, hDDIWK and rΔ10) were generated to further define the binding interface with Nef. These peptides were also biotinylated at the conserved cysteine residues mentioned above. Biotinylation was carried out for 2 hours at room temperature and pH 6.5 to 7.0 with a 10-fold molar excess of maleimide-PEG_2_-Biotin (Pierce), followed by reverse-phase HPLC purification using a C18 Vydac 4.6×250 mm analytical column (Vydac, Hesperia, CA) with linear acetonitrile gradient (0–72%) in 0.1% TFA (1 mL/min). Fractions containing the biotinylated peptides were identified by mass-spectrometry, pooled and lyophilized.

### Recombinant Nef protein expression and purification

A nearly full-length SIV_mac_239 Nef protein (residues 4–263), the core domain (residues 96–237) and different Nef deletion mutants lacking residues in the flexible loop were expressed in *E. coli* BL21 (DE3) as 6-His-thioredoxin fusion proteins and purified as described previously by Sigalov et al. [54]. After cell lysis with phosphate/Tris buffered 8 M urea solution (pH 8), the fusion protein was purified by affinity chromatography using NiNTA (Qiagen) under denaturing conditions (8 M urea), and refolded by dialysis against 20 mM Tris pH 8.0, 150 mM NaCl, 0.1 mM DTT. The soluble fusion protein was digested with thrombin (MP Biochmicals) leaving both the full-length protein and the core domain of SIV_mac_239 with two additional N-terminal residues (GS). Further purification was performed by anion-exchange chromatography (POROS 20 HQ, Applied Biosystems) and size-exclusion chromatography (Superdex 200, GE Healthcare).

### Surface plasmon resonance

Surface plasmon resonance experiments were carried out on a BIAcore 3000 instrument at 25°C. Neutravidin (approximately 30,000 resonance units (RU)) were coupled to a CM5 sensor chip (GE Healthcare) in 10 mM acetate buffer pH 5.0, and 0.005% (v/v) surfactant P20 at 10 µl/min using standard amine coupling protocols. Excess activated dextran carboxylate groups were capped with ethanolamine. Biotinylated peptides of tetherin (rBST-2_1–26_ and hBST-2_1–21_) were captured (3,000 RU) in different neutravidin-coupled experimental flow cells leaving one flow cell unmodified as a neutravidin-only control surface. Nef binding was studied at 25°C in PBS under reducing conditions (5 mM DTT). Purified samples of full-length, core domain or flexible loop deletion mutants of SIV_mac_239 Nef protein were injected at a flow rate of 5 µl/min over each experimental and control flow cell generating SPR sensorgrams. The sensorgram from the control cell was subtracted from the sensorgram of each experimental flow cell to correct for any nonspecific interaction with the CM5 or neutravidin surface. No regeneration step was required. Experiments were run in triplicate. For equilibrium analysis RU binding levels at equilibrium were extrapolated from each sensorgram (corrected for nonspecific interaction) in the concentration series, and plotted against concentration to derive a binding curve that was fit to a hyperbolic equation y = RU_max_*x/(K_d.app_+x), where y is the observed RU value, x is the concentration of Nef, and adjustable parameter RU_max_ and K_d.app_ are the RU value at saturation and the apparent binding constant (K_d.app_), respectively.

### MHC class I and CD4 downregulation in transfected Jurkat cells

Ten million Jurkat cells were electroporated with bicistronic pCGCG constructs (40 µg) that express wild-type Nef, or Nef mutants, and green fluorescent protein (GFP) from a downstream internal ribosomal entry site. Twenty-four hours later, cells were stained with a PerCP-conjugated monoclonal antibody to CD4 (BD Pharmingen, Billerica, MA) and an APC-conjugated monoclonal antibody to MHC-I (HLA-ABC, Dako, Carpintería, CA). After gating on the GFP^+^ cells, the mean fluorescence intensity (MFI) of CD4 and MHC I expression was determined. Data was collected using a FACSCalibur flow cytometer (Becton Dickenson) and analyzed using FlowJo 8.8.7 software (TreesStar).

### Infectivity enhancement assays

293T cells were co-transfected with *nef*-deleted SIV proviral DNA (100 ng) and either pCGCG, pCGCG-Nef or pCGCG-Nef mutants (100 ng each). All transfections were performed in duplicate in 24-well plates seeded the day before at 5×10^4^ cells per well, using GenJet Lipid Transfection Reagents (SignaGen Laboratories, Gaithersburg, MD). Forty-eight hours post-transfection, the amount of virus released into the cell culture supernatant was measured by SIV p27 antigen-capture ELISA (Advanced Bioscience Laboratories, Inc., Kensington, MD). Next, 50 ng of p27 equivalents for each virus were inoculated overnight onto GHOST X4/R5 cells seeded the day before in 12-well plates at 2.5×10^4^ cells per well. Twenty-four hours later, cells were washed and kept in fresh media. Forty-eight hours post-infection, cells were fixed and analyzed by flow cytometry as described above. The amount of infected cells was determined by calculating the percentage of GFP^+^ cells, and the infectivity of each Nef mutant relative to wild-type Nef was determined.

### Tetherin downregulation assays

293T cells stably expressing HA-tagged rhesus tetherin (5×10^4^ cells) were transfected with 200 ng of pCGCG-Nef (or Nef mutants) or empty vector. In the case of experiments with dominant-negative mutants of endocytic pathways, cells were also transfected with 300 ng of each of the expression vectors coding for the dominant-negative mutants or empty vectors. Twenty-four hours post-transfection, cells were briefly trypsinized and stained for the surface expression levels of tetherin with a primary mouse monoclonal anti-HA antibody (Covance, Princeton, NJ) at a dilution of 1∶4 and a secondary donkey anti-mouse APC-conjugated antibody (BD Pharmingen, Billerica, MA) at a dilution of 1∶40. Cells were gated on the GFP^+^ population and the levels of tetherin at the cell surface were determined by calculating the MFI. The percentage of tetherin present at the plasma membrane was calculated by dividing the MFI obtained in each transfection by the MFI obtained in transfections with empty vectors. Data was collected using a FACSCalibur flow cytometer (Becton Dickenson) and analyzed using FlowJo 8.8.7 software (TreesStar).

### Virus replication curves with Dynasore

Two million 221 T cells, a *Herpesvirus saimiri*-immortalized rhesus macaque CD4^+^ T cell line [47], were infected in duplicate with 20 ng p27 of SIV_mac_239 and SIV_mac_239 Δ*nef*. After 3 h of incubation at 37°C, cells were washed three times and resuspended in 5 ml of R20+IL-2 (100 U). One day post-infection cells were treated with 100 U of IFNα, and 8 hours later one of the replicates was treated with 20 µM of Dynasore. Virus replication was monitored at selected time points by p27 antigen-capture ELISA of the culture supernatant.

### Confocal microscopy

293T cells stably expressing HA-tagged rhesus macaque tetherin (2×10^4^ cells in a 8-well slide) were infected with VSV-G pseudotyped SIV_mac_239 Δ*env* (50 ng p27 eq.). Twenty-four hours later, cells were washed and fixed for 10 minutes in acetone/methanol and blocked for 20–60 minutes with 100 mM glycine diluted in 10% normal goat serum in PBS with 0.2% fish skin gelatin, 0.1% Triton ×100 and 0.02% sodium azide (10% NGS-PBS-FSG-Tx100-NaN_3_). The cells were then washed three times in 10% NGS-PBS-FSG-Tx100-NaN_3_, and stained. The mouse monoclonal antibodies 17.2 (IgG_1_) and 3H4 (IgG_2a_) were used at a dilution of 1∶250 to stain for Nef and tetherin, respectively. The cells were subsequently stained with Alexa-488- and Alexa-568-conjugated goat anti-mouse secondary antibodies specific for IgG_1_ and IgG_2a_, respectively (Invitrogen, Grand Island, NY) (1∶1000), and with TO-PRO3 (Invitrogen) (1∶5000) to visualize cell nuclei. To stain intracellular compartments, rabbit polyclonal antibodies specific for TGN46 (Sigma-Aldrich, St Louis, MO), CD63 (Santa Cruz Biotechnology, Santa Cruz, CA) and LAMP-1 (abcam, Cambridge, MA) were used at a dilution of 1∶50. Next, an Alexa-568 goat anti-rabbit (Invitrogen, Grand Island, NY) was used to detect these cellular markers. In this case, Nef staining was performed by using a secondary Alexa-633-conjugated goat anti-mouse IgG_1_. After staining, the cells were washed and mounted on slides with antiquenching mounting-medium (Vector Laboratories, Inc). Images were acquired using a Leica TCS SP5 II confocal microscope.

## Supporting Information

Figure S1 **Comparison of protein expression in cell lysates to the accumulation of SIV p27 in the cell culture supernatant.** Western blots were performed to compare protein levels in cell lysates and virions resulting from transfections with Nef mutants with impaired anti-tetherin activity. Membranes were developed with antibodies specific for tetherin, p55 Gag, p27 CA, Nef, tetherin and β-actin.
(TIF)

Figure S2 **Analysis of Nef proteins with deletions in the flexible loop region for binding to rhesus tetherin and TCRζ.** Representative SPR traces for the binding of Nef_96–237_ (A) and Nef_96–237_ Δ197–205 (B) to a peptide corresponding to the cytoplasmic domain of rhesus tetherin. Representative SPR traces for the binding of Nef_96–237_ (C) and Nef_96–237_ Δ197–205 (D) to a peptide corresponding to residues 65–80 of the TCRζ chain cytoplasmic domain. (E) Estimated K_d_ values for the binding of recombinant SIV Nef_96–237_ proteins with the indicated deletions in the flexible loop region to a peptide corresponding to residues 65–80 of the TCRζ chain peptide.
(TIF)

Figure S3 **Analysis of tetherin peptides for direct binding to Nef.** Representative SPR traces for the binding of Nef_96–237_ and rhesus tetherin (A), a tetherin mutant lacking 10 amino acids (B), a rhesus tetherin mutant containing alanine substitutions at positions 14–18, (C) and a human tetherin mutant containing residues D_14_DIWK_18_ from rhesus tetherin (D).
(TIF)

Figure S4 **CD4-downregulation by SIV Nef mutants with impaired anti-tetherin activity.** (A–D) Jurkat cells were electroporated with bicistronic constructs expressing each of the SIV Nef mutants and GFP. Cells were stained with a PerCP-conjugated monoclonal antibody to CD4 and the MFI of CD4 staining, indicated in the upper right corner of each plot, was determined after gating on the GFP^+^ cell population.
(TIF)

Figure S5 **MHC class I-downregulation by SIV Nef mutants with impaired anti-tetherin activity.** (A–D) Jurkat cells were electroporated with bicistronic constructs expressing each of the SIV Nef mutants and GFP. Cells were stained with an APC-conjugated anti-HLA class I-specific monoclonal antibody and the MFI of MHC class I staining, indicated in the bottom right corner of each plot, was determined after gating on the GFP^+^ cell population.
(TIF)

Figure S6 **Identification of residues in SIV Nef required for infectivity enhancement.** The infectivity of SIV Δ*nef trans*-complemented with Nef mutants containing alanine substitutions at the indicated positions was determined using GHOST X4/R5 cells. Virus was produced by co-transfecting 293T cells with SIV_mac_239 Δ*nef* proviral DNA, a construct expressing wild-type or mutant Nef, or empty vector (pCGCG). GHOST X4/R5 cells were infected with 50 ng of p27 equivalents of each virus, and the percentage of infected GFP^+^ cells was determined by flow cytometry 48 hours after infection. The relative infectivity of SIV Δ*nef trans*-complemented with mutants in the N-terminal domain (A), the globular core domain (B), and the flexible loop region (C) is shown in comparison to SIV Δ*nef trans*-complemented with wild-type Nef (black) and without *trans*-complementation (white). The black dotted lines indicate 5 standard deviation above the infectivity observed for SIV Δ*nef* without *trans*-complementation. (D) Nef mutants with impaired infectivity enhancement (below 5 standard deviations of SIV Δ*nef* activity) were tested for binding to Dyn2 by co-immunoprecipitation. 293T cells were co-transfected with expression constructs for the indictated Nef mutants, and either Dyn2-GFP or an empty vector (V). Cell lysates were immunoprecipitated with a monoclonal antibody to GFP and western blots were probed with antibodies to Nef and GFP. The ratios of the band intensities for Nef versus Dyn2 in the immunoprecipitated samples are shown beneath each lane.
(TIF)

Figure S7 **Subcellular distribution of tetherin in uninfected cells.** 293T cells expressing HA-tagged rhesus tetherin were stained for tetherin (HA) (green), Nef (red) and either TGN46, CD63 or LAMP-1 (blue). The white scale bar indicates 25 µm.
(TIF)
