# Supplementary figures and images for: Tetherin/BST-2 Antagonism by Nef Depends on a Direct Physical Interaction between Nef and Tetherin, and on Clathrin-mediated Endocytosis
Source: PLoS Pathog. 2013 Jul 11;9(7):e1003487. doi: 10.1371/journal.ppat.1003487 (PMC3708871; doi:10.1371/journal.ppat.1003487)

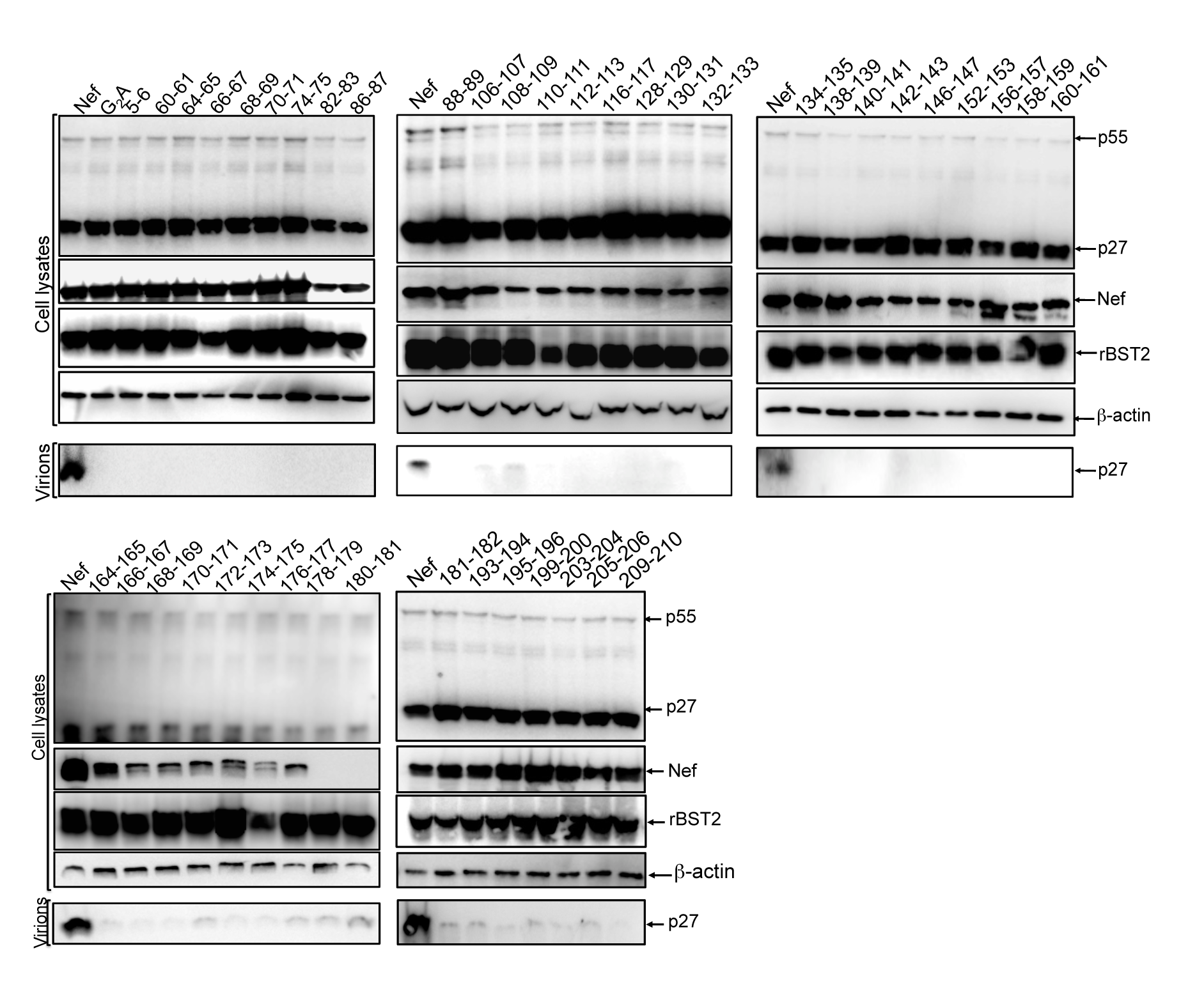

Supplement: Figure S1 — Comparison of protein expression in cell lysates to the accumulation of SIV p27 in the cell culture supernatant. Western blots were performed to compare protein levels in cell lysates and virions resulting from transfections with Nef mutants with impaired anti-tetherin activity. Membranes were developed with antibodies specific for tetherin, p55 Gag, p27 CA, Nef, tetherin and β-actin. (TIF) [file ppat.1003487.s001.tif]

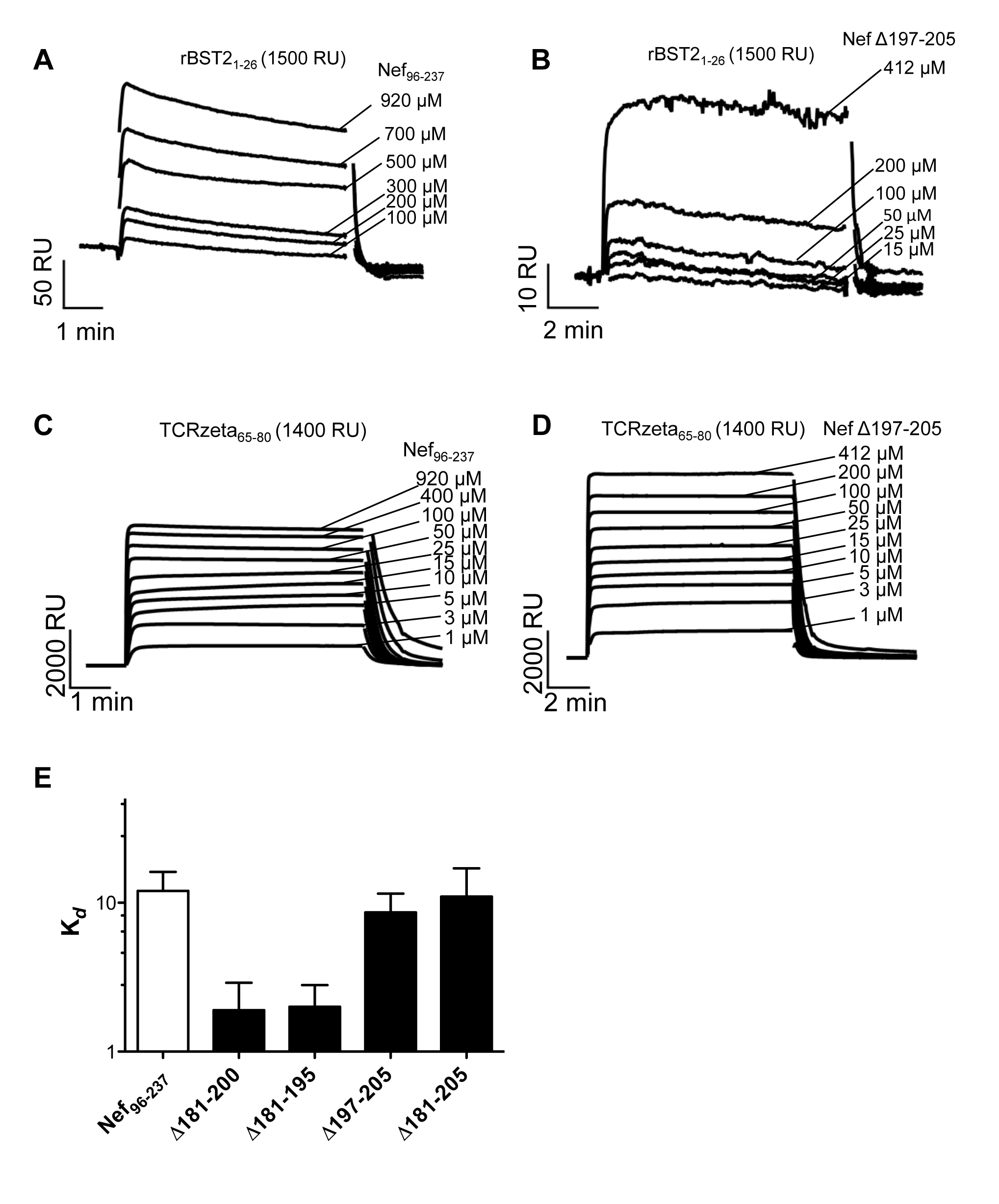

Supplement: Figure S2 — Analysis of Nef proteins with deletions in the flexible loop region for binding to rhesus tetherin and TCRζ. Representative SPR traces for the binding of Nef96–237 (A) and Nef96–237 Δ197–205 (B) to a peptide corresponding to the cytoplasmic domain of rhesus tetherin. Representative SPR traces for the binding of Nef96–237 (C) and Nef96–237 Δ197–205 (D) to a peptide corresponding to residues 65–80 of the TCRζ chain cytoplasmic domain. (E) Estimated Kd values for the binding of recombinant SIV Nef96–237 proteins with the indicated deletions in the flexible loop region to a peptide corresponding to residues 65–80 of the TCRζ chain peptide. (TIF) [file ppat.1003487.s002.tif]

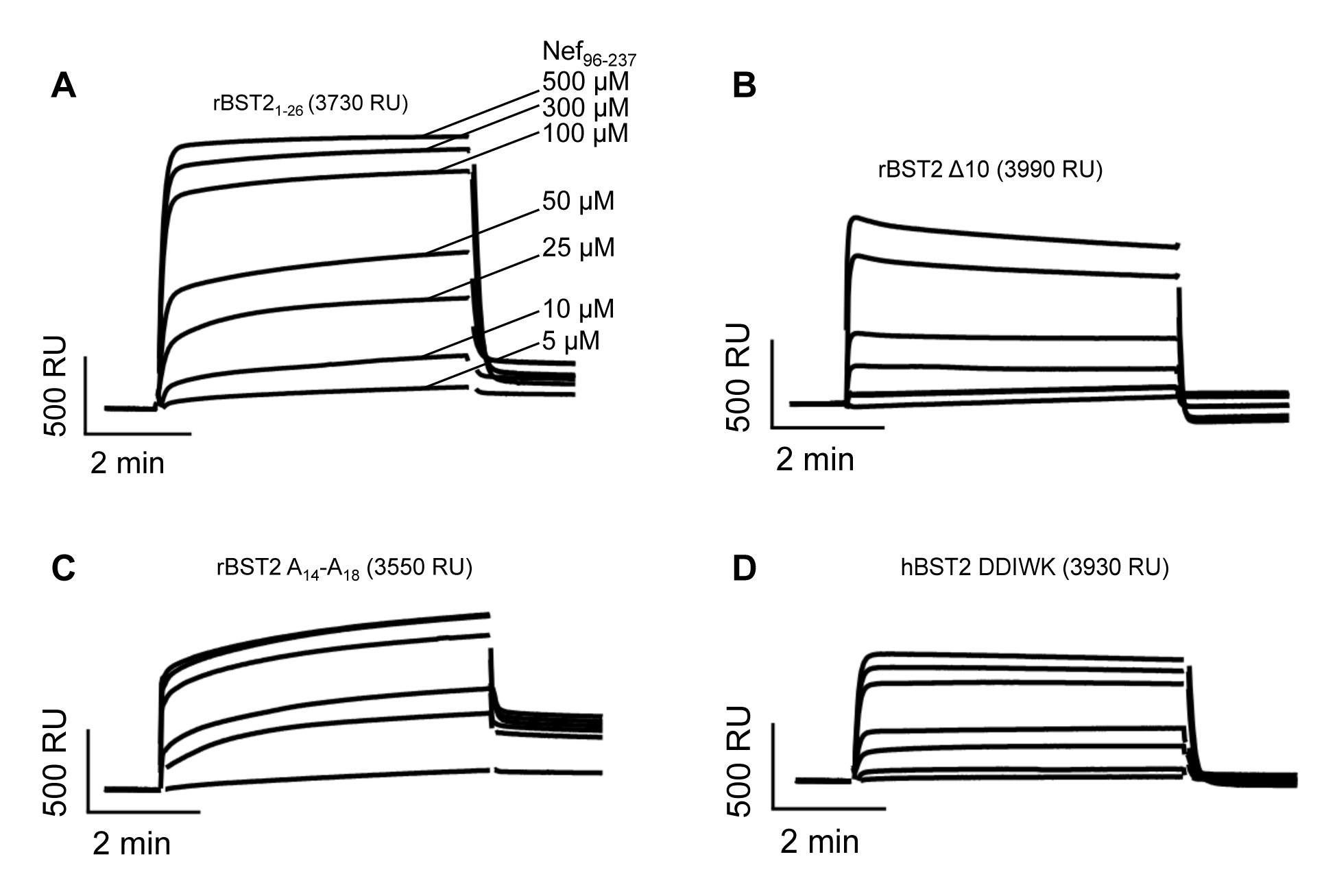

Supplement: Figure S3 — Analysis of tetherin peptides for direct binding to Nef. Representative SPR traces for the binding of Nef96–237 and rhesus tetherin (A), a tetherin mutant lacking 10 amino acids (B), a rhesus tetherin mutant containing alanine substitutions at positions 14–18, (C) and a human tetherin mutant containing residues D14DIWK18 from rhesus tetherin (D). (TIF) [file ppat.1003487.s003.tif]

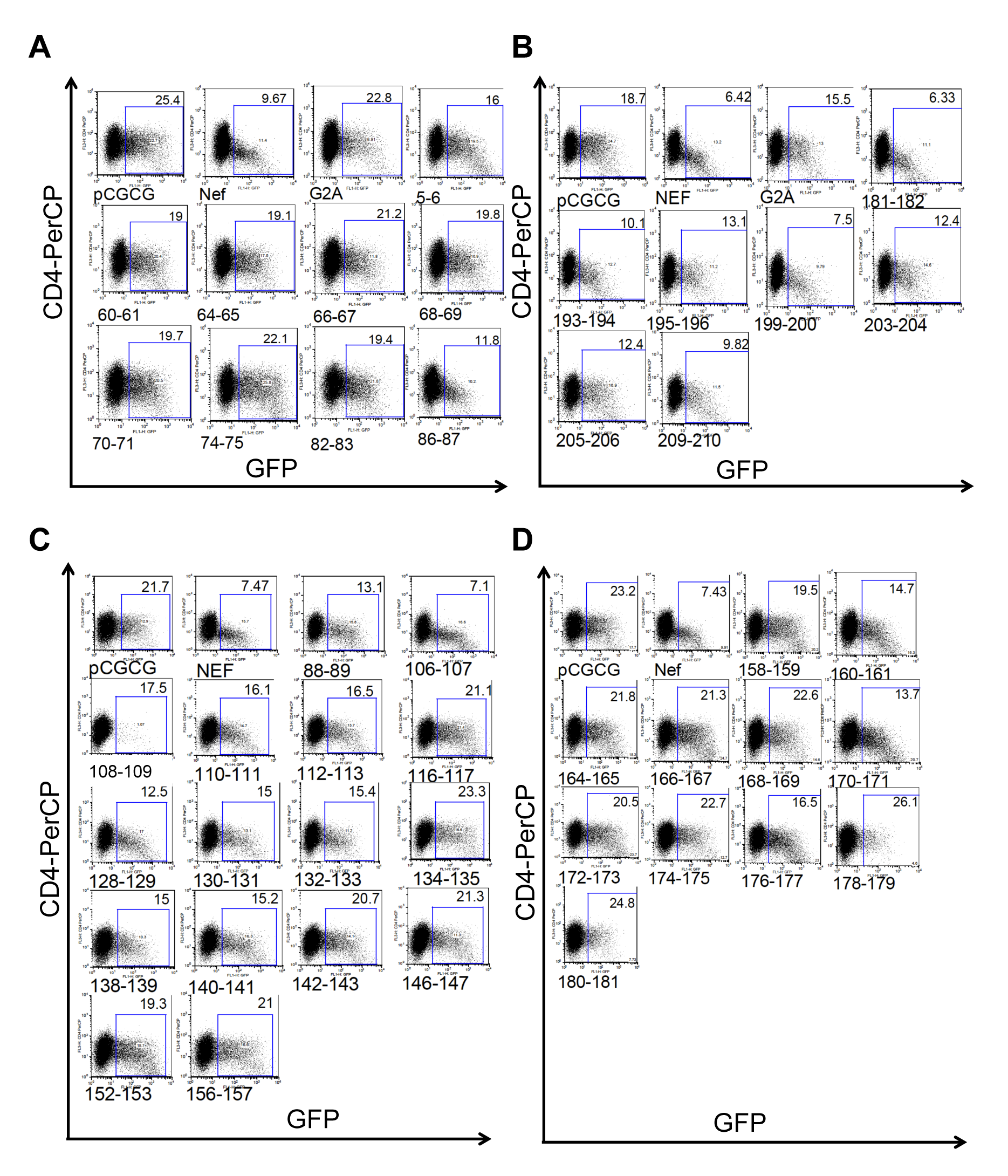

Supplement: Figure S4 — CD4-downregulation by SIV Nef mutants with impaired anti-tetherin activity. (A–D) Jurkat cells were electroporated with bicistronic constructs expressing each of the SIV Nef mutants and GFP. Cells were stained with a PerCP-conjugated monoclonal antibody to CD4 and the MFI of CD4 staining, indicated in the upper right corner of each plot, was determined after gating on the GFP+ cell population. (TIF) [file ppat.1003487.s004.tif]

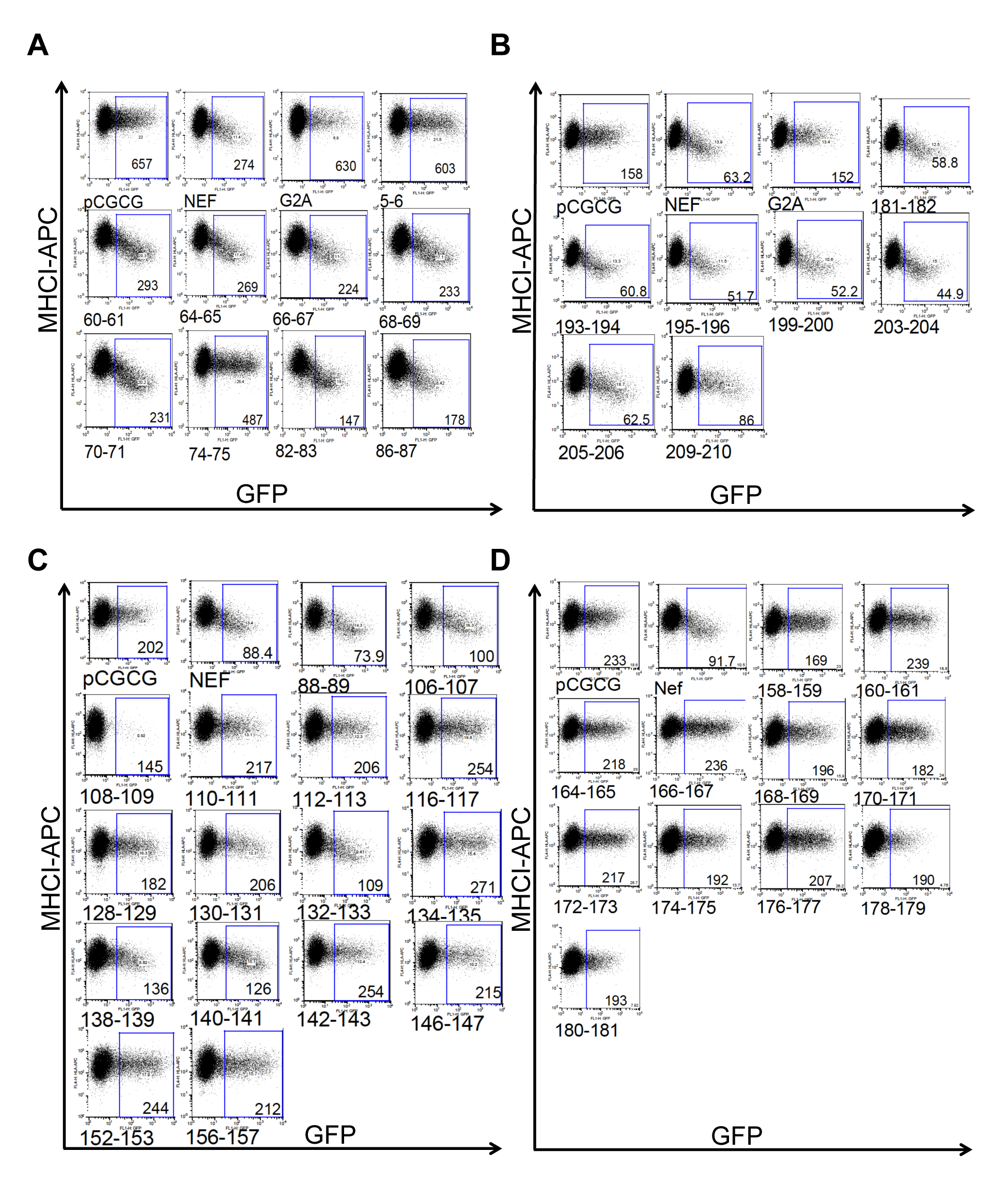

Supplement: Figure S5 — MHC class I-downregulation by SIV Nef mutants with impaired anti-tetherin activity. (A–D) Jurkat cells were electroporated with bicistronic constructs expressing each of the SIV Nef mutants and GFP. Cells were stained with an APC-conjugated anti-HLA class I-specific monoclonal antibody and the MFI of MHC class I staining, indicated in the bottom right corner of each plot, was determined after gating on the GFP+ cell population. (TIF) [file ppat.1003487.s005.tif]

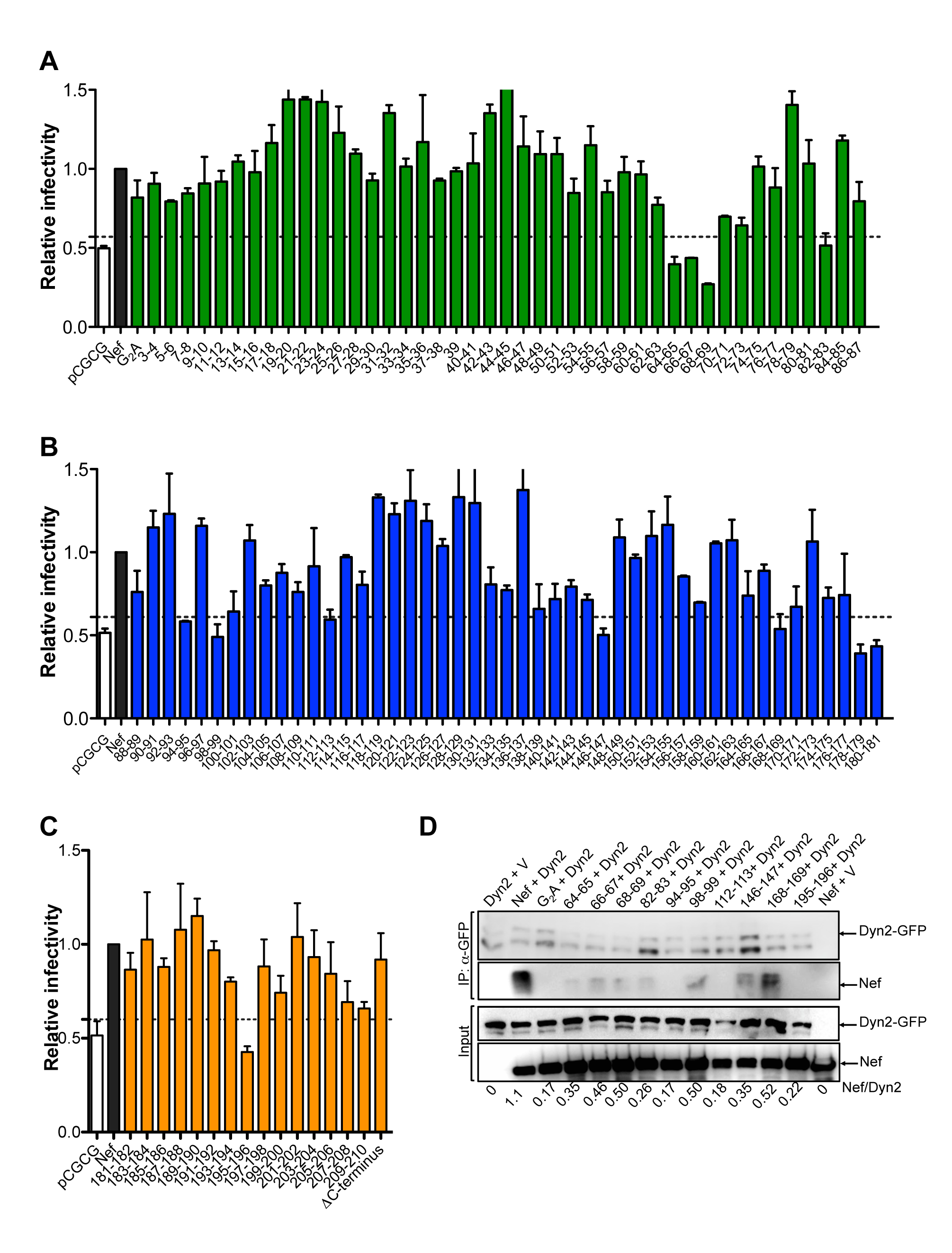

Supplement: Figure S6 — Identification of residues in SIV Nef required for infectivity enhancement. The infectivity of SIV Δnef trans-complemented with Nef mutants containing alanine substitutions at the indicated positions was determined using GHOST X4/R5 cells. Virus was produced by co-transfecting 293T cells with SIVmac239 Δnef proviral DNA, a construct expressing wild-type or mutant Nef, or empty vector (pCGCG). GHOST X4/R5 cells were infected with 50 ng of p27 equivalents of each virus, and the percentage of infected GFP+ cells was determined by flow cytometry 48 hours after infection. The relative infectivity of SIV Δnef trans-complemented with mutants in the N-terminal domain (A), the globular core domain (B), and the flexible loop region (C) is shown in comparison to SIV Δnef trans-complemented with wild-type Nef (black) and without trans-complementation (white). The black dotted lines indicate 5 standard deviation above the infectivity observed for SIV Δnef without trans-complementation. (D) Nef mutants with impaired infectivity enhancement (below 5 standard deviations of SIV Δnef activity) were tested for binding to Dyn2 by co-immunoprecipitation. 293T cells were co-transfected with expression constructs for the indictated Nef mutants, and either Dyn2-GFP or an empty vector (V). Cell lysates were immunoprecipitated with a monoclonal antibody to GFP and western blots were probed with antibodies to Nef and GFP. The ratios of the band intensities for Nef versus Dyn2 in the immunoprecipitated samples are shown beneath each lane. (TIF) [file ppat.1003487.s006.tif]

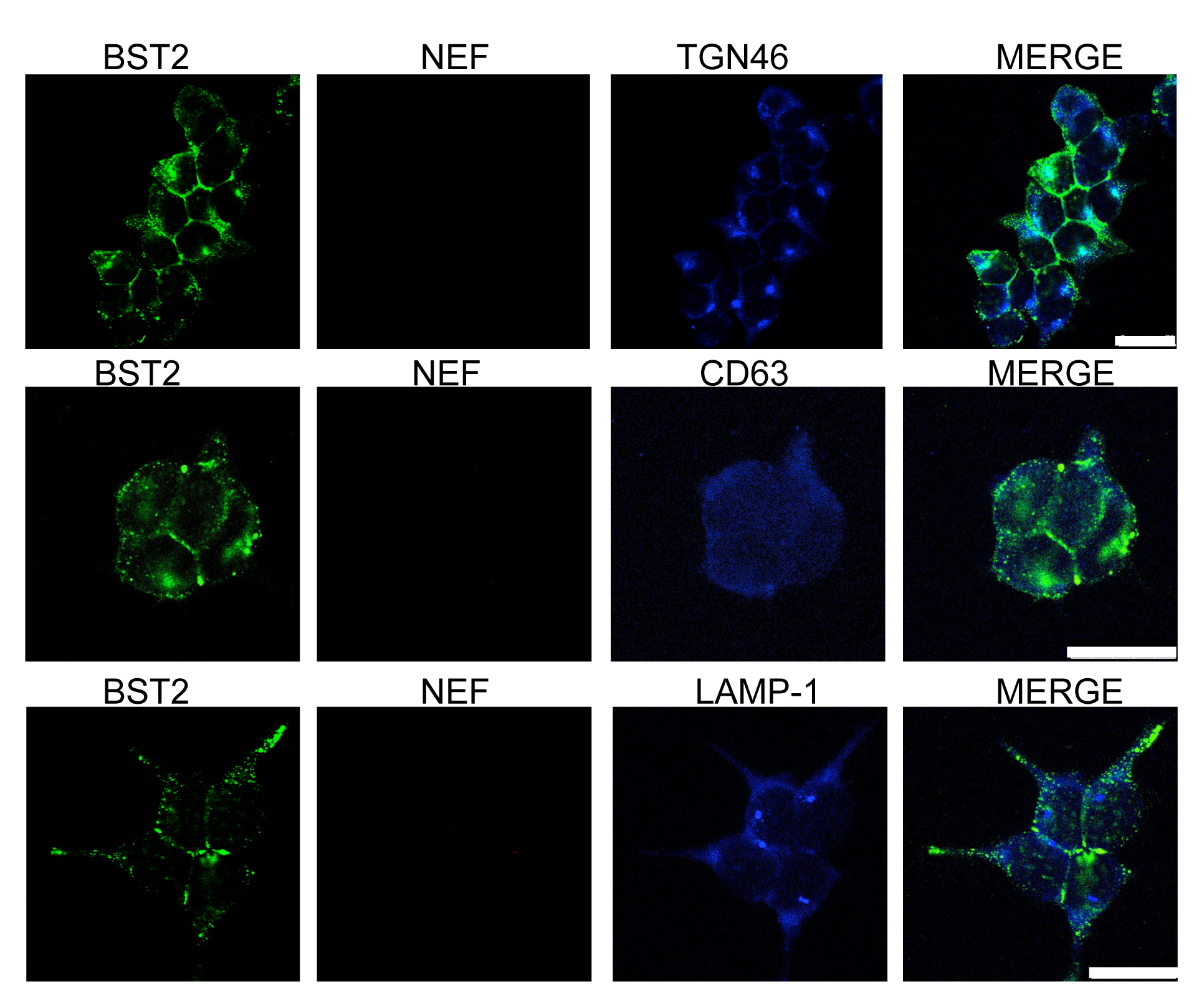

Supplement: Figure S7 — Subcellular distribution of tetherin in uninfected cells. 293T cells expressing HA-tagged rhesus tetherin were stained for tetherin (HA) (green), Nef (red) and either TGN46, CD63 or LAMP-1 (blue). The white scale bar indicates 25 µm. (TIF) [file ppat.1003487.s007.tif]
